# Thiol–Michael Addition to a Quinolin‐2‐One–Isoxazole Dye in Cationic CTABr Micellar Media: Insights From Experiments and Theory

**DOI:** 10.1002/open.70275

**Published:** 2026-08-09

**Authors:** Guillermo E. Quintero, Kevin Droguett, Costantino Zazza, Margarita E. Aliaga

**Affiliations:** ^1^ Facultad de Química y de Farmacia Escuela de Química Pontificia Universidad Católica de Chile Santiago Chile; ^2^ Department of Organic Faculty of Chemistry and Biology, Chemistry Universidad de Santiago de Chile Santiago Chile; ^3^ Department for Innovation in Biological, Agro‐food and Forest systems Università della Tuscia (DIBAF) Viterbo Italy

**Keywords:** computational chemistry, fluorescent probe, surfactants, sustainable chemistry, UV‐visible spectra

## Abstract

Herein we report an experimental and theoretical study of the reactivity of the dye (E)‐7‐(diethylamino)‐1‐methyl‐3‐(2‐(3‐methyl‐4‐nitroisoxazol‐5‐yl)vinyl)quinolin‐2‐one (**DQI**) toward bisulfite (HSO_3_^−^) in the presence of an aqueous micellar solution of the cationic surfactant cetyltrimethylammonium bromide (CTABr), which was used as the reaction medium. We observe that aqueous **DQI** exhibits very weak emission, accompanied by a low fluorescence quantum yield. Interestingly, upon addition of CTABr, a pronounced enhancement of the fluorescence intensity is observed, together with a hypsochromic shift in the emission. Additionally, the cationic surfactant acts as an efficient reaction promoter by enhancing the solubility of the proposed hydrophobic **DQI** dye and increasing its local concentration within the Stern layer, where bisulfite ions are also accumulated, thereby favoring a nucleophilic attack. The formation of thiol adducts is investigated via photophysical (absorption and fluorescence) and nuclear magnetic resonance measurements and the derived data are further supported by density functional theory‐correlated wavefunctions to identify the most favorable sites for electrophilic and nucleophilic attack in the **DQI** probe. The overall evidence clearly indicates a distinctive reactivity, supporting the formation of a **DQI•**SO_3_H Michael adduct in the proposed CTABr micellar medium.

## Graphical abstract

We report the reactivity of **DQI** toward bisulfite in CTABr micelles. CTABr enhances **DQI** fluorescence and promotes the reaction by increasing the solubility and local concentration. Spectroscopy, NMR, and DFT support the formation of a **DQI•**SO_3_H Michael adduct and identify preferred reactive sites.

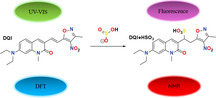

## 1 Introduction

**FIGURE 1:**
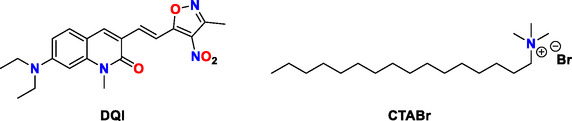
Chemical structures of dye (*E*)‐7‐(diethylamino)‐1‐methyl‐3‐(2‐(3‐methyl‐4‐nitroisoxazol‐5‐yl)vinyl)quinolin‐2‐one (**DQI**) and the cationic cetyltrimethylammonium bromide (CTABr) surfactant employed in this work.

A green strategy aimed at improving water solubility is the use of cationic surfactants, such as cetyltrimethylammonium bromide (CTABr) (see chemical structure in Figure 1), which have been shown to enhance the solubility of fluorescent probes when their concentration exceeds a threshold known as the critical micelle concentration (CMC), forming structures called micelles [1]. In this way, the newly formed aqueous microenvironment, which differs from neat water in its apolar character, improves probes solubility and also affects the photophysical properties of guest molecules upon incorporation into the micellar interface, known as the Stern layer, through supramolecular interactions. These mutual interactions are generally governed mainly by hydrophobic interactions, with binding constants (*K*_s_) of the order of 10^3–^10^4^ M^−1^ [2, 4]. Thanks to this balance of mutual effects, aqueous micellar media nowadays provide a green solution for expanding the use of fluorescent probes in water, thereby increasing their applicability to various analytes in aqueous matrices [5, 6]. Several fluorescent probes have been reported in the literature. Among them, quinolin‐2(1*H*)‐one is highlighted as a biologically active core, with antifungal, anticancer, and antibiotic properties [7, 9]. Their use has gained interest in different research areas due to their stability, aromaticity, and potential applications [10, 11]. Researchers face synthetic challenges, particularly in achieving adequate yields of these compounds while making water‐soluble probes [12]. However, in practice, most fluorescent probes are poorly soluble in aqueous media and may require the use of cosolvents, such as ethanol (EtOH), acetonitrile (ACN), and dimethyl sulfoxide (DMSO), among others [13]. Previously, our group reported a quinolin‐2‐one dye based on nitroisoxazole moiety, which exhibited poor water solubility [14]. We found that the use of a cationic micellar medium, specifically CTABr, significantly enhanced the dye’s solubility in water and, in parallel, increased the Michael‐type reaction rate with bisulfite (HSO_3_^−^) by 200‐fold. This demonstrated not only improved solubility in aqueous media but also enhanced reactivity toward nucleophilic analytes. Additionally, to further demonstrate the applicability of this core as a fluorescent probe for HSO_3_^−^ detection, we reported a novel quinolin‐2(1*H*)‐one chalcone‐based fluorescent probe. In a CTABr cationic micellar medium, this probe efficiently detected bisulfite with a low limit of detection (LOD) in the micromolar range (0.7 μM). Moreover, it was successfully applied to real white wine samples, demonstrating its potential for practical applications [15].

Interestingly, both quinolinone derivatives mentioned above share the ability to interact with the bisulfite anion through a Michael‐type addition, enabled by the presence of a sufficiently electrophilic C═C double bond. This reactivity promotes bisulfite addition and induces pronounced changes in UV–Vis and fluorescence spectra. Similar behavior has been reported for other fluorophore cores, such as coumarins [16], xanthenes [17], quinolines [18], among others. Of relevance is the role of the isoxazole moiety, and more specifically, the nitroisoxazole group, in finely tuning the reactivity of fluorescent probes toward bisulfite. In this respect, it has been demonstrated that the presence of this moiety adjacent to a carbon–carbon double bond substantially increases the electrophilicity of the carbon *α*‐unsaturated relative to the fluorophore core, resulting in significantly shorter reaction times in practice [19]. Based on these considerations, it is compelling to evaluate the reactivity of a fluorescent core, such as a 7‐(diethylamino)quinolin‐2(1*H*)‐one derivative bearing a nitroisoxazole moiety conjugated via a C═C double bond, toward bisulfite in a CTABr aqueous micellar solution.

With this target in mind, herein we investigate the Michael‐type addition between the dye (*E*)‐7‐(diethylamino)‐1‐methyl‐3‐(2‐(3‐methyl‐4‐nitroisoxazol‐5‐yl)vinyl)quinolin‐2‐one (**DQI**) (Figure 1) and bisulfite in an aqueous micellar solution of CTABr. More specifically, using a combination of experimental and theoretical studies, we investigate how the micellar environment affects both the reactivity and photophysical properties of **DQI** compared to those in an aqueous solution. This integrated approach provides insight into the role of micellar media in modulating electrophilic activation and spectral response in bisulfite sensing systems.

## 2 Experimental and Theoretical Section

### 2.1 Materials and Methods

All reagents and solvents used in this work, including deuterated ones, were purchased from Merck and employed as received. All solutions were prepared freshly and **DQI** was synthesized according to the literature, and all the analytical data matched those reported [20]. Nuclear magnetic resonance (NMR) spectra were obtained at 25 °C on a Bruker Avance 400 MHz spectrometer and were processed with MestreNova v14.2 software. The Michael addition reaction was followed by mixing appropriate volumes of **DQI** solutions and bisulfite in acetonitrile (ACN), ACN‐d_3_, and water‐d_2_ (D_2_O).

Solutions of **DQI** in DMSO and surfactant in aqueous solutions were prepared daily. The absorption spectra were recorded on a double‐beam UV–Vis spectrophotometer model DS5 from Edinburgh Instruments, UK. The fluorescence spectrum was obtained by using a Cary Eclipse fluorescence spectrometer from Agilent Technologies. They were recorded at temperatures of 25.0 ± 0.1  and 27.0 ± 0.1 °C, depending on the case, using quartz cuvettes with an optical path of 1 cm. The CMC value was obtained by fluorescence spectroscopy, exciting at the maximum absorption wavelength of **DQI** at a concentration of 2.2 μmol L^−1^. The concentration of the stock solution for CTABr was 0.116 M. Aqueous dilutions of CTABr were prepared from 0.09 to 1.4 mM with an increment of 0.09 mM from the stock solution. By plotting the fluorescence intensity at the maximum wavelength against the stoichiometric surfactant concentration, the CMC can be determined by observing the change in slope at the point where the two tangent lines intersect.

In parallel, the binding constants (*K*_s_) were obtained by fluorescence spectroscopy, exciting at the maximum absorption wavelength of **DQI** at a concentration of 2.2 μmol L^−1^. The value of *K*_s_ has been determined using the nonlinear regression method [Equation (1) ] [21]

(1) $$I=\;\frac{I_{w}^{0}+\;I_{m}^{0}K_{s}(C_{s}-\mathrm{CMC})}{1+\;K_{s}(C_{s}-\mathrm{CMC})}$$

where *I* is the fluorescence intensity of **DQI** in the presence of surfactants, subscripts w and m indicate aqueous and micellar pseudophases, respectively, $I_{w}^{0}$ and $I_{m}^{0}$ correspond to the respective intensity of **DQI** in the aqueous and micellar pseudophases, *C*_s_ is the total surfactant concentration, and CMC is the critical micellar concentration.

Subsequently, the fluorescence quantum yield of **DQI** in water and aqueous micellar solution of CTABr were measured using coumarin 153 in ethanol as standard (*φ*_s_ = 0.53) [22] according to [Equation (2)]

(2) $$\varphi_{x}=\varphi_{s}\left(\frac{{\mathrm{Grad}}_{x}}{{\mathrm{Grad}}_{s}}\right)\left(\frac{n_{x}^{2}}{n_{s}^{2}}\right)$$

where φ is the quantum fluorescent yield, subscripts *x* and s indicate sample (**DQI** solutions in this case) and standard, respectively, *n* is the refractive index, and *Grad* is the slope from the plot of integrated fluorescence intensity versus absorbance. Additionally, fluorescence decays were measured using a Lifespec II picosecond fluorescence lifetime spectrometer from Edinburgh Instruments, UK. The experimental conditions used in this work were carried out according to the report by Zuñiga‐Núñez et al. [23]. The values were obtained using an excitation source of 506.2‐nm laser diode (EPL‐510, with an full width at half maximum (FWHM) of 88 ps). The maximum number of counts collected to determine fluorescence lifetimes was 10,000 counts. The emission was collected at the wavelength corresponding to the fluorescence maximum of **DQI** in water and aqueous micellar solution of CTABr. The IRF was deconvoluted with fluorescence decays to obtain fluorescence lifetimes.

### 2.2 Computational Details

At first, we started optimizing the molecular structure of the selected **DQI** probe in a polarizable and continuum media modeling the encircling aqueous environment via the popular Conductor‐like polarizable continuum model (C‐PCM) [24]. More specifically, the theoretical level employed in this study was the density functional theory (DFT) [25] adopting two different hybrid generalized gradient approximation functionals for the exchange–correlation energy: the Becke 3‐parameters Lee–Yang–Parr (B3LYP) functional [26, 28]; the parameter‐free PBE0 functional that mixes 25% Hartree–Fock exact exchange with 75% Perdew–Burke–Ernzerhof (PBE) exchange and 100% PBE correlation [29]. Moreover, based on a previous investigation on a similar system, a Def2‐TZVP valence triple‐zeta polarization Karlsruhe atomic basis set was adopted [30]. Also, we used Grimme’s D3(BJ) dispersion correction for the van der Waals (vdW) interactions correction [31, 33]. Furthermore, with the target of characterizing the measured **DQI** photophysical properties (see Section 2.1), the lowest valence UV–Vis absorption bands of such a chromophore were estimated by using the time‐dependent DFT (TD‐DFT) approach [34]. The vertical absorption bands are then estimated by combining the emerging vertical excitation energies with oscillator strengths and using Gaussian broadening functions having a FWHM of 3000 cm^−1^ (i.e., the Δ*v′*_1/2_) [35]. The formula used to convolute the computed TD‐DFT[C‐PCM/(B3LYP or PBE0)] UV–Vis absorption spectra is given below

(3) $$\varepsilon \left(v\prime \right)=\frac{2.175\;\cdot 10^{8}}{\text{Δ}v_{\frac{1}{2}}^{\prime }}\cdot f\;\cdot \exp\left[-2.772\;\cdot \frac{{\left(v\prime -\;v_{i\to f}^{\prime }\right)}^{2}}{\text{Δ}v_{\frac{1}{2}}^{\prime }}\right]$$

where *f* (the oscillator strength under the transition dipole length approximation) and *v′*_*i → f*_ (the vertical excitation energy in wavenumbers, cm^−1^) are both directly extracted from TD‐DFT calculations. The oscillator strength is related to the transition dipole moment, *M*_*i→f*_ by the standard equation

(4) $$f_{i\to f}=\frac{8\;\pi \;m_{e\;}}{3\;e^{2\;}h^{2}}\cdot \text{Δ}E_{i\to f}\cdot \;{\left|M_{i\;\to \;f}\right|}^{2}=\;\frac{8\;\pi \;m_{e\;}c}{3\;e^{2\;}h^{2}}\cdot v_{i\;\to \;f}^{'}\cdot \;{\left|M_{i\;\to \;f}\right|}^{2}$$

where *m*_e_ and e are the mass and the charge of the electron, respectively, *h* is Planck’s constant, and *c* is the speed of light. The energy difference (Δ*E*_*i *→* f*_) between these states is related to the frequency *v*, the wavelength *λ*, and the wavenumber *vʹ* of absorbed light by the equation



(5)

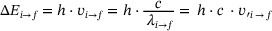




The results of TD‐DFT calculations are then convoluted using Gaussian‐type curves to create the associated UV–Vis spectra; more specifically, each electronic transition is associated with a Gaussian curve according to the expression of the *ε*(*vʹ*) value which provides an estimation of the molar absorption coefficient, in M^−1^ cm^−1^, as reported in Equation (3) [35, 36].

In addition, solvation pathways surrounding **DQI** solute in aqueous solution were also investigated via classical molecular dynamics technique. More in detail, the previously obtained **DQI** structure—at the C‐PCM(H_2_O)/PBE0/Def2‐TZVP level—was used to generate a classical all‐atom atomic force field; in this respect, bond, angle, dihedral, and Lennard–Jones OPLS‐AA parameters were extracted from LigParGen web‐based service provided by the Jorgensen group [37], initial charges were estimated using the molecular electrostatic potential method of Merz–Kollman [38], and all bonds involving hydrogen atoms were constrained using the LINCS algorithm [39]. As a next step, the solvation dynamics of aqueous **DQI** was analyzed by carrying out a 200‐ns molecular dynamics (MD) simulations at 298 K in an NVT ensemble with the solute placed at the center of a cubic box with an edge of 46 Å; solvent molecules were treated according to the SPC scheme [40] and MD simulations were performed using GROMACS Software (release 2022.6) [41]. In this respect, we also have a look at the reservoir of noncovalent interactions (NCIs) at play within 4.0 Å from the molecular probe via the recently developed amIGM method [42], a computational technique used to analyze and visualize weak intermolecular interactions within MD trajectories. The associated algorithm, as implemented in Multiwfn toolbox [43, 44], calculates time‐averaged, electron‐density‐based maps to reveal interaction strengths and locations in dynamic environments, offering high image quality and lower computational costs compared to other methods [42].

As a conclusive step, we also investigated at DFT, the **DQI•**SO_3_H adduct arising from a Thiol–Michael addition to the **DQI** molecule with local reactivity descriptors in the field of the conceptual DFT (C‐DFT) framework [45]. In particular, we have characterized electrophilic and nucleophilic regions of **DQI** by using the empirically defined orbital‐weighted Fukui functions [$f_{w}^{-}(r)$ and $f_{w}^{+}(r)$] [46] as defined by the following expressions

(6) $$f_{w}^{-}\left(r\right)=\sum_{i}^{\mathrm{HOMO}}w_{i}\rho_{i}\left(r\right);\;f_{w}^{+}\left(r\right)=\sum_{\mathrm{LUMO}}^{\infty }w_{i}\rho_{i}\left(r\right)$$

in which *w*_*i*_ are the so‐called weighting factors, *ρ*_*i*_(***r***) represents the square of the *i‐*th molecular orbital, and the term *i* runs over a set of MOs. The *w*_*i*_ terms are defined—for both the $f_{w}^{-}(r)$ and $f_{w}^{+}(r)$ functions—according to the Gaussian ansatz as follows

(7) $$w_{i}=\frac{\exp\;\left[-{\left(\frac{\mu -\varepsilon_{i}}{\text{Δ}}\right)}^{2}\right]}{\sum_{i}^{\mathrm{HOMO}}\exp\;\left[-{\left(\frac{\mu -\varepsilon_{i}}{\text{Δ}}\right)}^{2}\right]};\;w_{i}=\frac{\exp\;\left[-{\left(\frac{\mu -\varepsilon_{i}}{\text{Δ}}\right)}^{2}\right]}{\sum_{\mathrm{LUMO}}^{\infty }\exp\;\left[-{\left(\frac{\mu -\varepsilon_{i}}{\text{Δ}}\right)}^{2}\right]}$$

where *µ* is the chemical potential—estimated as (*E*_HOMO_ + *E*_LUMO_)/2)—$\varepsilon_{i}$ the energy of each molecular orbital and Δ represents the width of a Gaussian‐type function (set to 0.1 Hartree) determining the energy range for orbitals that make a substantial contribution to the reactivity [47]. In particular, when the **DQI•**SO_3_H adduct is concerned, a subset of different conformations are optimized at PBE0(D3(BJ))/Def2‐TZVP level of computation considering such a system encircled by 1‐IodoHexaDecane solvent according to C‐PCM model [24]; this solvent was selected from those available in the Gaussian 16 code (release C.01) [48] to approximate a CTABr micellar medium. In addition, we also investigated an intramolecular hydrogen bonding contact between HSO_3_^−^ moiety and a local carbonyl group of the **DQI** species by coupling the quantum theory of atoms in molecule (QTAIM) [49] with the graphic representation of the local electron energy density (GLED) model recently proposed in the literature by one of us (CZ) [50, 51]. The method relies on plotting the GLED, the *H*(***r***), to generate 3D isosurfaces, the *H*(0, ISO), which allows to visually distinguish between covalent, noncovalent, and ionic bond characteristics. This method was highlighted as a way to provide a simple, visual interpretation of bonding phenomena within complex chemical systems at DFT cost.

## 3 Results and Discussion

### 3.1 Photophysical and Computational Characterization of DQI

**FIGURE 2:**
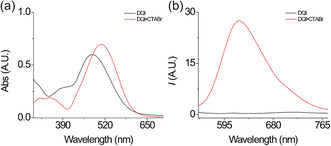
(a) UV–Vis absorption spectrum of **DQI** (26 μM) and (b) emission spectrum of **DQI** (2.2 μM) in water and CTABr (10 mM). *λ*_ex_ = 511 nm; slit, 10 nm/10 nm.

The photophysical properties of **DQI** were investigated. In aqueous solution, **DQI** exhibited limited solubility; however, concentrations of up to 1 μM were achieved by adding 1% DMSO as a cosolvent. Under these conditions, **DQI** displayed an absorption maximum at 474 nm at time zero, in good agreement with our previously reported values [20]. In contrast, when **DQI** was dissolved in an aqueous micellar solution of CTABr (10 mM), a pronounced bathochromic shift of ≈40 nm was observed, yielding a new absorption maximum at 511 nm. A comparison of the absorption spectra in both media is shown in Figure 2a. This redshift was accompanied by a significant increase in the molar absorptivity coefficient (*ε*). In water, *ε* was determined to be 3.2 × 10^4^ M^−1^ cm^−1^, whereas in the micellar medium, it increased to 5.1 × 10^4^ M^−1^ cm^−1^, representing a 1.6‐fold enhancement relative to water. These results are shown in Table 1. The observed bathochromic shift and enhanced absorptivity are attributed to the nonpolar microenvironment provided by the micellar core, where the long hydrocarbon chains of CTABr create a markedly less polar environment compared to bulk water.

**TABLE 1:** Photophysical properties of **DQI** in water and aqueous micellar solutions of CTABr.

Surfactant	*λ*_abs_, nm	*λ*_em_, nm	*ε* a, 10^4^ M^−1^ cm^−1^	$\varphi_{f}$	CMC^b^, mM	*K*_s_ c, 10^3^ M^−1^	*τ*_1_ (ns)/A_1_	*τ*_2_ (ns)/A_2_	*τ*_3_ (ns)/A_3_	<*τ*>
Water	474	689	3.2 ± 0.05	0.0004	—	—	n.d.	n.d.	n.d.	—
CTABr	511	620	5.1 ± 0.05	0.0044	0.754	19.3 ± 0.97	0.02(86%)	0.28(9%)	0.74(5%)	0.08

a Values obtained at *λ*_max_ of absorption at 25 °C.

b Value obtained at 27 °C.

c Values obtained by fluorescence excitation at *λ*_max_ of the dye at 27 °C.

**FIGURE 3:**
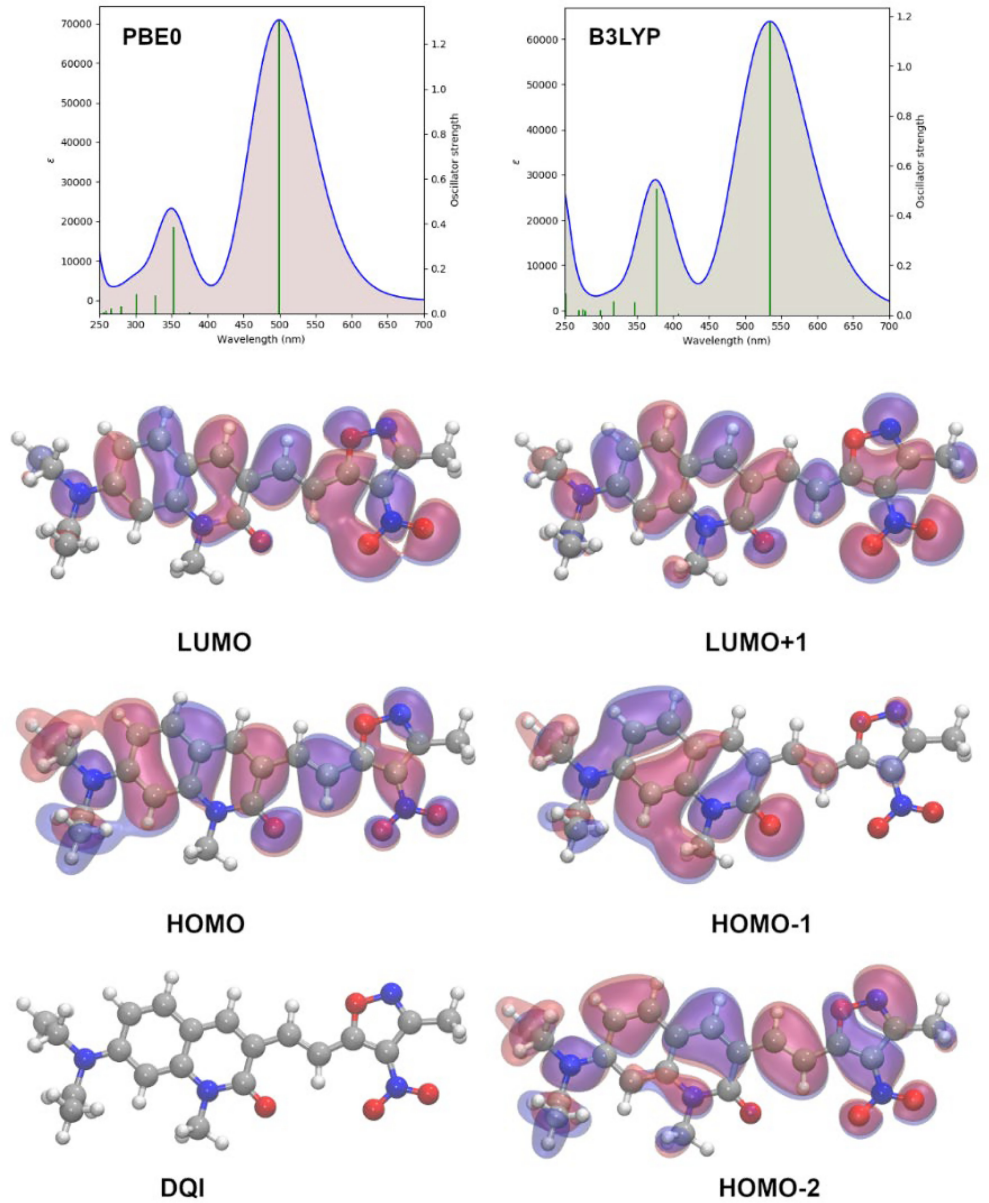
Upper panel: UV–Vis absorption spectra of **DQI** at C‐PCM(H_2_O)/PBE0//B3LYP[D3(BJ)]/Def2‐TZVP level. The molar absorption coefficient (*ε*) is reported in M^−1^ cm^−1^. Profiles are obtained by combining excitation energies with oscillator strengths and using Gaussian broadening functions with a FWHM of 3000 cm^−1^; oscillator strengths are reported as green histograms. Lower panel: HOMO (*π*) and LUMO (π*) orbitals—at C‐PCM/PBE0 level—as extracted from the optimized structure of the **DQI** responsible for transitions with oscillator strength >0.15; the blue color reflects the positive part of the corresponding eigenvector; an isodensity value of 0.015 *e*·*a*_0_^−3^ is used.

On the other hand, the time‐dependent variation of the maximum absorption wavelength of **DQI** in water and in aqueous micellar solution of CTABr was kinetically monitored (see Figures S1 and S2). The kinetic profile of **DQI** in aqueous solution containing 1% DMSO showed that, within the first 5 min, the absorbance at 474 nm began to decrease, and over a total period of 8 h, the characteristic purple coloration of the solution gradually became colorless, with an overall decrease in the absorbance of ≈40% (see Figure S1). This change in absorbance was accompanied by the formation of a dark purple precipitate, similar in color to **DQI** **,** suggesting that the compound slowly precipitates over time. Furthermore, due to the presence of a labile C═C double bond susceptible to nucleophilic attack, **DQI** would be readily hydrolyzable; therefore, the observed decrease in absorbance is attributed to cleavage of the conjugation between the quinolinone and nitroisoxazole moieties. In contrast, in the presence of a 10 mM aqueous micellar solution of CTABr, the absorbance of **DQI** at 511 nm remained unchanged over 16 h, with no significant variation in the kinetic profile (see Figure S2). The absence of absorbance changes indicates that no disruption of the **DQI** conjugation occurs, and no precipitate formation was observed. In view of these results, the presence of a micellar medium enhances the solubility of the dye and also inhibits hydrolysis and/or decomposition of the dye by incorporation into the Stern layer of CTABr micelles. This phenomenon has been previously reported in the literature [52, 55], and our research group has earlier described the stabilization of a quinolinone chalcone based on the presence of CTABr [15]. Always remaining in this context, the estimated UV–Vis spectrum of **DQI** solute in water within the TD‐DFT formalism is shown in Figure 3, upper panel. Looking at this figure, it is clear that, within the imposed simulation environment, the B3LYP functional tends to overestimate the position of the lowest energy absorption band; as a matter of fact, a value of 534 nm (*f* = 1.18) is predicted. On the other hand, the parameter‐free PBE0 functional providing a value of 499 nm (*f* = 1.30) is seen to slightly overestimate the absorption detected at 474 nm (see Table 1). Moreover, in line with the literature, such a band arises from a HOMO→LUMO electronic excitation featuring a valence ^1^ *π* → π* character (see Figure 3). Again, the absorption band estimated at almost 350 nm instead results from electronic transitions mainly involving HOMO→LUMO + 1, HOMO‐1→LUMO, and HOMO→LUMO + 2 electronic excitations. All these orbitals feature a *π* → π* character during the excitation pathways widespread along the entire aromatic skeleton of the **DQI** chromophore. The only exception is represented by the HOMO‐1 molecular orbital, which is substantially confined in the left‐side delocalized aromatic region (see Figure 3).

For the sake of completeness, it is worth noting that always within the imposed TD‐DFT framework, when the **DQI** molecular probe is embedded in a micellar media simulated via a 1‐IodoHexaDecane solvent according to C‐PCM model [24], the expected blue shift with respect to the aqueous media, as shown in Table 1 and Figure 2a, is not observed (see Figure S3). On the other hand, a *λ*_max_ at 511 nm (*f* = 1.20) in line with that measured in the laboratory is obtained when introducing a model **DQI/**CTA^+^ supramolecular assembly in a water dielectric media (Figure S4). In this respect, the mutual position of the CTA^+^ cation was selected on the basis of the electrostatic surface potential of the investigated chromophore, as shown in Figure S5.

**FIGURE 4:**
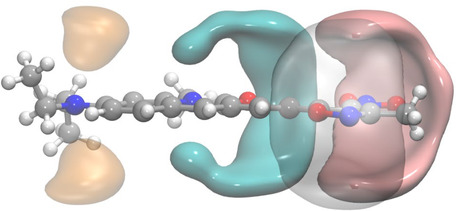
Water density regions featuring solvent molecules at H‐bond contact with the **DQI** solute. These isosurfaces [isodensity value of 0.05 (HW_1_ or HW_2_) Å^−3^] are derived by analyzing a classical MD trajectory of 40 ns carried out considering **DQI** in a water simulation box at 298 K and pressure of 1 atm. Please note that different colors reflect different interacting moieties of the solute.

**FIGURE 5:**
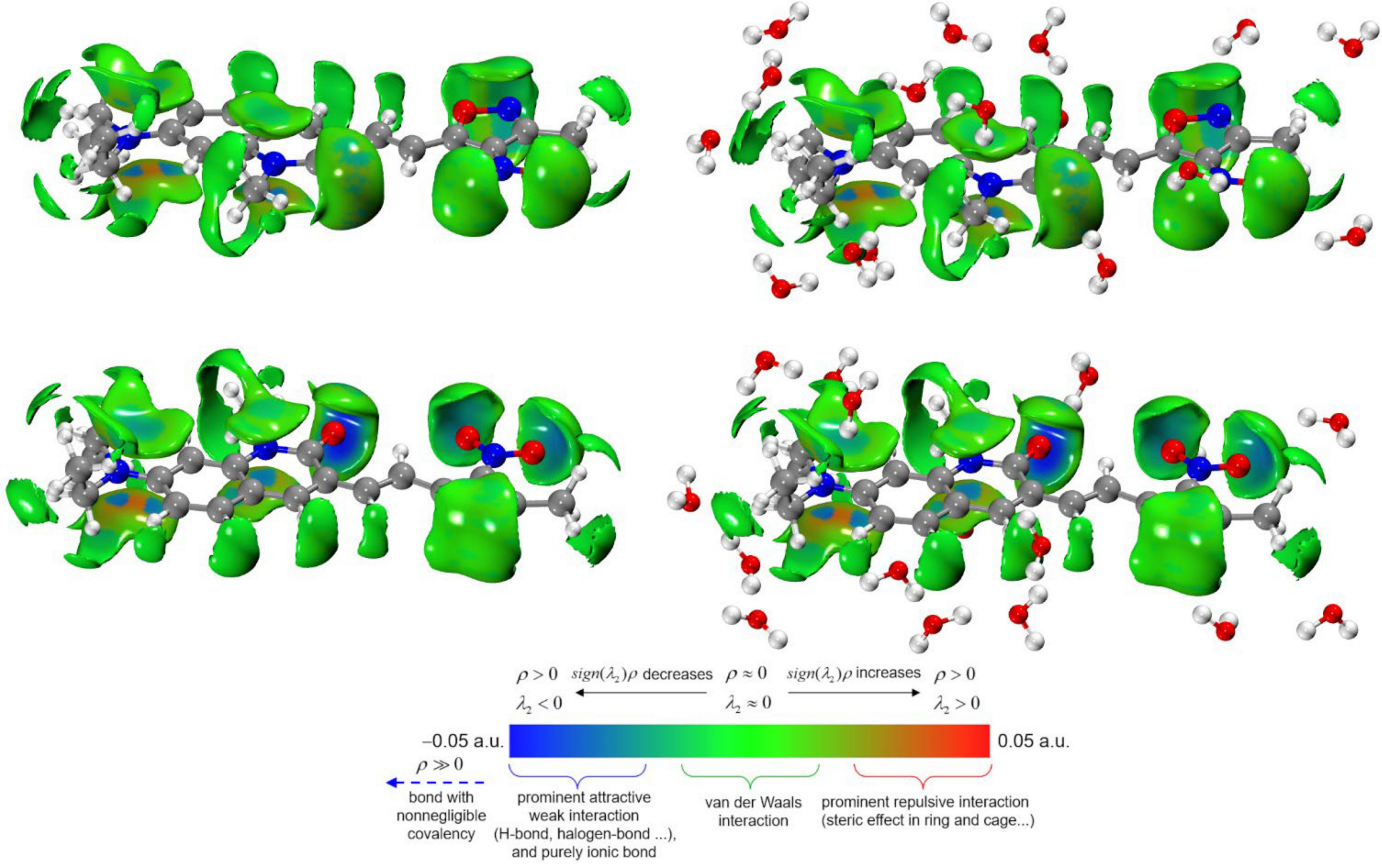
Average NCIs between the solvated **DQI** molecular probe and the surrounding aqueous environment revealed by amIGM algorithm. We used a classical MD simulation of 40 ns, 2000 frames, a probe shape box of 4.0 Å, and a grid spacing of 0.20 *a*_0_. The maps correspond to an isovalue of $\delta {\bar{g}}^{\text{inter}}$ equal to 0.002; the coloring method used is also shown. We also overimposed water molecules within 4.0 Å from the solute as sampled in a generic MD frame.

The nanosolvation pathways encircling the **DQI** solute in water are also addressed using a 40‐ns MD simulation at finite room temperature condition. More specifically, we focused our attention on water molecules within 4.0 Å from the solute and, using a frame every 20 ps, we estimated the distribution of the solvent around **DQI**, and the result is shown in Figure 4. Looking at this figure, it is rather evident that H‐bond acceptor groups coordinate water molecules within well‐defined wet surfaces encircling them. Please note that in this analysis, we used a different color for each functional group. Interestingly, we also observe that the diethylamino (─NEt_2_) substituent features water molecules in the vicinity of the nitrogen atom despite the presence of the two ethyl groups. In turn, local nanosolvation pathways are investigated using the amIGM algorithm [42], as implemented in Multiwfn toolbox [43, 44], to perform a visual analysis of weak interactions involved along the 40 ns of the MD trajectory. In this method, detectable interfragment interactions are revealed by isosurfaces of averaged *δg* ^inter^ ($\delta {\bar{g}}^{\text{inter}}$), which is simply the time average of the *δg* ^inter^(*t*) calculated for every frame in a MD trajectory written in XYZ format. The averaged sign(*λ*_2_ *ρ*) is used to color the isosurfaces of $\delta {\bar{g}}^{\text{inter}}$, which is evaluated with the same way as a NCIs, [42, 56] namely *ρ*(*r*) corresponds to time averaged promolecular density—spherically averaged built‐in free‐atom density model—and *λ*_2_ corresponds to the second largest eigenvalue of time averaged Hessian of promolecular density; as a result, the computational cost of such an analysis is linearly proportional to the number of considered frames in the MD trajectory [42]. Furthermore, before applying the amIGM analysis, the MD trajectory resulting from the Gromacs software [41] was centered with respect to the **DQI** molecule and, for each frame, a least‐squares fit was applied to align the classical frames. The estimated maps in two different orientations are shown in Figure 5. The isosurfaces, estimated considering 2000 MD frames, and along with the overimposed mapped sign(*λ*_2_ *ρ*) colors in the amIGM analysis perfectly reveal the regions of the molecular solute that are mainly engaged in NCIpatterns modulated by thermal effects with the surrounding aqueous environment. Thus, Figure 4 shows the most recurring positions sampled by solvent molecules within the solute/solvent interface during the dynamics, while Figure 5 shows a picture of the mutual supramolecular interactions averaged over the entire trajectory. As expected, the distribution of the local amIGM isosurfaces—isovalue of $\delta {\bar{g}}^{\text{inter}}$ = 0.002—results in line with the **DQI** nanosolvation pathways observed. The nitro group within the 3‐methyl‐4‐nitro isoxazol moiety is seen to act as a strong, noncovalent H‐bond acceptor as well as the carbonyl group of the quinolin‐2‐one skeleton. This trend can be appreciated by looking at the confined blue region of the plotted interfacial amIGM isosurfaces. A slightly weaker H‐bond electrostatic interactions are also observed for the O and N atoms in the five‐membered ring constituting the isoxazol five‐membered ring and for the N atom of one of the aromatic rings of the quinolin‐2‐one group; in this respect, also the H‐bond patterns sampled around the ─NEt_2_ group are clearly revealed on average on the basis of the imposed all‐atoms force field. The remaining depicted green isosurfaces represent regions in which, within the explored time range, the mutual solute/solvent interactions can be substantially characterized as weak vdW interactions of variable nature (e.g., dispersion, stacking interactions) [42]. Finally, we would like to mention that interfacial water molecules encircling the **DQI** probe in a generic classical MD frame are also visualized in the same figure.

Regarding the fluorescence spectra, **DQI** exhibits very weak emission at 689 nm, accompanied by a low fluorescence quantum yield ($\varphi_{f}$ = 0.0004), as shown in Figure 2b and Table 1. Interestingly, upon addition of CTABr, a pronounced enhancement of the fluorescence intensity is observed, together with a hypsochromic shift of the emission maximum to 620 nm (≈70 nm blue shift). As a consequence, the $\varphi_{f}$ increases to 0.0044, corresponding to an enhancement of approximately one order of magnitude. It is noteworthy that our group previously reported that **DQI** exhibits inverse solvatochromic fluorescence behavior, which depends exclusively on the solvent’s proton‐donating or proton‐accepting nature [20]. This represents an anomalous case, as **DQI** is the second organic molecule reported in the literature to exhibit such behavior. In this context, a 10 mM CTABr solution exhibits a $E_{30}^{T}$ value of 54.4 kcal/mol at 25 °C and a $E_{N}^{T}$ value of 0.73 [57]. This polarity is consistent with the observed hypsochromic shifts in fluorescence upon increasing solvent polarity, mimicking the spectral behavior typically observed in nonpolar solvents. Furthermore, **DQI** contains a ─NEt_2_ at position 7 of the quinolin‐2‐one core, which has been extensively studied as an excellent electron‐donating group that enhances electronic delocalization within the dye [58, 61]. This favors intramolecular charge transfer (ICT) states, thereby increasing the fluorescence of various dyes, as this transition state is highly emissive [62, 63]. However, it is well known that this group can undergo torsion, disrupting the planarity of the molecule and generating a twisted intramolecular charge transfer (TICT) state, which is typically nonemissive [64, 65]. As a result, dyes in general in polar protic solvents, such as water, exhibit low $\varphi_{f}$. In the case of **DQI**, this behavior is observed when the dye is in aqueous media with a low $\varphi_{f}$. However, in the presence of CTABr, an increase in the $\varphi_{f}$ is observed when the dye is inserted into the micelle. This is most likely due to the fact that, within the Stern layer, the microviscosity of this region is significantly higher compared to that of neat water, preventing the ─NEt_2_ group from twisting and thereby inhibiting the formation of the TICT state, while promoting emissive states like ICT [66].

To further elucidate the excited‐state behavior, fluorescence lifetimes measurements were performed and are shown in Table 1. It is important to note that **DQI** undergoes aggregation in aqueous media, preventing reliable lifetime measurements under these conditions. In contrast, in CTABr micellar environments, the fluorescence decay profiles could be satisfactorily resolved into three distinct lifetime components. These were assigned to the locally excited (LE), ICT, and TICT states, corresponding *τ*_1_, *τ*_2_, and *τ*_3_, respectively. Notably, the dominant contribution of the LE state is consistent with the photophysical behavior previously reported for quinolin‐2(1*H*)‐one derivatives, supporting the assignment and reinforcing the role of restricted intramolecular motion within the organized micellar medium [67].

### 3.2 Determination of Critical Micelle Concentration (CMC) and Binding Constants (*K*_s_) by Fluorescence

**FIGURE 6:**
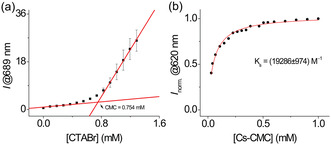
(a) Relationship between the fluorescence intensity at the emission peak 689 nm of **DQI** and the concentration of CTABr. (b) Fitting the fluorescence intensity normalized values to determine the probe−micelle association constant for **DQI** in CTABr aqueous micellar solution. Errors in CMC and *K*_s_ are less than 10%.

To study the influence of CTABr concentration on the photophysical properties of **DQI**, it is essential to determine the CTABr’s CMC under our experimental conditions. In previous experiments, the CMC of CTABr was assumed to be ≈1 mM [68] and measurements were carried out under conditions that ensured micellar systems, typically at concentrations about ten times higher than the CMC. However, to ensure accurate interpretation of subsequent studies, determining the CMC under the specific experimental conditions employed here is of paramount importance. For this purpose, changes in the fluorescence band of **DQI** in water (see Table 1) were monitored at different CTABr concentrations, both below and above the theoretical CMC, in order to identify a change in slope. Figure 6a shows a plot of the fluorescence intensity of **DQI** in water at 689 nm as a function of the stoichiometric concentration of CTABr. A clear change in slope is observed at a CTABr concentration of 0.754 mM. This value corresponds to the concentration at which CTABr monomers begin to form micelles under the experimental conditions used in this study. Notably, the experimentally determined CMC is ≈25% lower than the value reported in the literature. This deviation can be attributed to the presence of DMSO and to the measurement temperature of 27 °C, both of which are known to favor CTABr micellization at lower surfactant concentrations [1, 39]. The temperature of 27 °C was selected to prevent precipitation of CTABr, which occurs at ≈25 °C, known as the Krafft temperature [40]. A temperature as close as possible to this threshold was chosen to avoid precipitation while minimizing temperature‐induced spectroscopic variations. This consideration was consistently applied in all subsequent analyses throughout the study.

In addition, we determined the binding constant (*K*_s_) of **DQI•**CTABr using fluorescence spectroscopy. This analysis revealed that the fluorescence intensity of **DQI** increases as the micellar concentration of CTABr rises, as shown in Figure 6b. Notably, the micellar aqueous solution of CTABr exhibits a significantly higher fluorescence intensity when [Cs‐CMC] is zero. The relationship between fluorescence intensity and micellar concentration was fitted according to Equation (1), with the results shown in Table 1. Through data fitting, a *K*_s_ value of 1.93 × 10^4^ M^−1^ was obtained. The *K*_s_ values in the range of 10^4^ M^−1^ indicate a strong interaction between the isoxazole‐based quinolin‐2‐one derivative and the Stern layer of CTABr, conferring a nonpolar microenvironment. This supports the hydrophobic nature of **DQI** when incorporated into the micelle. Similar *K*_s_ values have been reported in literature. For instance, Kaur et al. [41] reported *K*_s_ values on the order of 10^3^ M^−1^ for a styryl dye with CTABr. More recently, Bandyopadhyay et al. [42] investigated the photophysical properties of eosin yellow with various surfactants, including cetyltrimethylammonium chloride (CTACl), and found a *K*_s_ value of 5.76 × 10^4^ M^−1^. Both studies suggest that the magnitude of *K*_s_ is governed by hydrophobic interactions between the dyes and the micellar medium. More specifically, our research group has reported *K*_s_ values like those of **DQI**, further confirming the hydrophobic nature of the dye under study [14, 15].

### 3.3 Experimental and Theoretical Evidence of Thiol–Michael Adduct Formation

**FIGURE 7:**
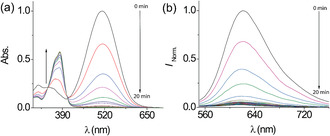
Time‐dependent (0–20 min) (a) absorption spectrum of **DQI** (26 μM) and (b) emission spectrum of **DQI** (2.2 μM) in the presence of 50 equiv. of HSO_3_^−^ in CTABr (10 mM) and PBS (10 mM) at pH 7.4 and 27 °C. *λ*_ex_ = 511 nm; slit, 10 nm/10 nm.

After conducting both experimental and theoretical studies to elucidate the photophysical behavior of **DQI** in the absence and presence of a micellar medium formed by CTABr, the influence of HSO_3_^−^ on the photophysical properties of **DQI** was investigated. Initially, the intense purple coloration of the solution was completely bleached upon the addition of 50 equivalents of bisulfite, a process that was monitored by UV–Vis spectroscopy. Figure 7a shows the time‐dependent absorbance spectra recorded in the presence of bisulfite. These experiments were carried out in an aqueous micellar solution of CTABr (10 mM) in PBS (10 mM, pH 7.4) at 27 °C. Within 20 min, the characteristic absorption band of **DQI** in CTABr, initially centered at 511 nm, disappeared, accompanied by the emergence of a new band at ≈380 nm. Simultaneously, an isosbestic point at 400 nm was observed, indicating a reaction between **DQI** and HSO_3_^−^ and supporting the formation of a new **DQI•**SO_3_H adduct. The appearance of the absorption band at 380 nm has been previously reported by our group for quinolin‐2‐one derivatives in the presence of HSO_3_^−^ and is attributed to the presence of the 7‐(diethylamino)quinolin‐2‐one core [15]. In parallel, fluorescence spectroscopy revealed a pronounced quenching of the **DQI** emission at 620 nm upon reaction with bisulfite, as shown in Figure 7b, which can be attributed to the disruption of the electronic conjugation within the molecular probe and the loss of molecular planarity.

**FIGURE 8:**
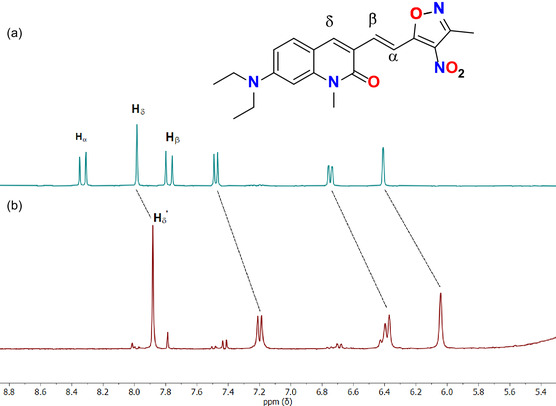
Partial ^1^H‐NMR of (a) **DQI** and (b) **DQI•**SO_3_H in CH_3_CN/D_2_O (1:2).

We have demonstrated that **DQI** undergoes UV–Vis discoloration, accompanied by a decrease in emission intensity, upon interaction with HSO_3_^−^. However, these observations do not provide direct structural insight into the site of HSO_3_^−^ addition. Literature precedents describe related systems in which HSO_3_^−^ adds via a Michael‐type reaction to an electrophilic carbon–carbon double bond linking two molecular fragments [15, 43, 45]. In **DQI**, such a double bond connects the quinolin‐2‐one and isoxazole moieties. Based on the above, carbon *β* (regarding of isoxazole moiety) is expected to be the most electrophilic, an effect further enhanced by the presence of the nitro group (see chemical structure in Figure 1), which acts as a strong electron‐withdrawing substituent and increases the lability of this carbon. To demonstrate this, ^1^H NMR spectra of **DQI** were recorded before and after the addition of HSO_3_^−^, and the comparative results are shown in Figure 8. It is important to note that CTABr could not be used in this experiment due to solubility issues that limited its applicability. Consequently, an ACN/D_2_O solvent mixture was employed to ensure adequate solubilization of both **DQI** and bisulfite.

**SCHEME 1:**
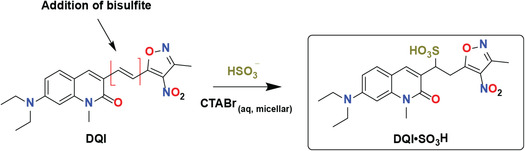
The proposed reaction site of **DQI** molecule with bisulfite.

Before the addition of bisulfite, the signals corresponding to protons of the carbons *α*, *β*, and *δ* of **DQI** can be observed at 7.81, 8.36, and 8.01 ppm, respectively. The signals at 7.81 and 8.36 ppm are assigned to the protons of the double bond connecting the two moieties of the molecule. This double bond exhibits a *trans* configuration, as evidenced by the large coupling constant (*J* = 16.1 Hz), as clearly shown in Figure 8a. Upon addition of HSO_3_^−^ to the sample and ≈2 min later, the spectra shown in Figure 8b reveal the disappearance of the signals corresponding to protons on carbons *α* and *β* of **DQI**, along with a slight upfield shift (≈0.1 ppm) of the proton on *C* *_δ_*. These observations indicate that the bisulfite addition occurs at the carbon–carbon double bond under discussion, rather than at the double bond within the quinolin‐2‐one core where the proton *δ* is located. This selectivity is reasonable, as reaction at the quinolin‐2‐one core would result in loss of aromaticity, which is unfavorable under these conditions. The signals of protons of *C* *_α_* and *C* *_β_* could not be clearly identified after the reaction due to overlap with nondeuterated ACN, whose resonances span the 4.5–6.0 ppm region where these protons are expected to appear. Nevertheless, the data clearly support the addition of bisulfite to **DQI**, resulting in the formation of the **DQI•**SO_3_H adduct. This adduct is shown in Scheme 1, which illustrates the proposed reaction of **DQI** with bisulfite.

**FIGURE 9:**
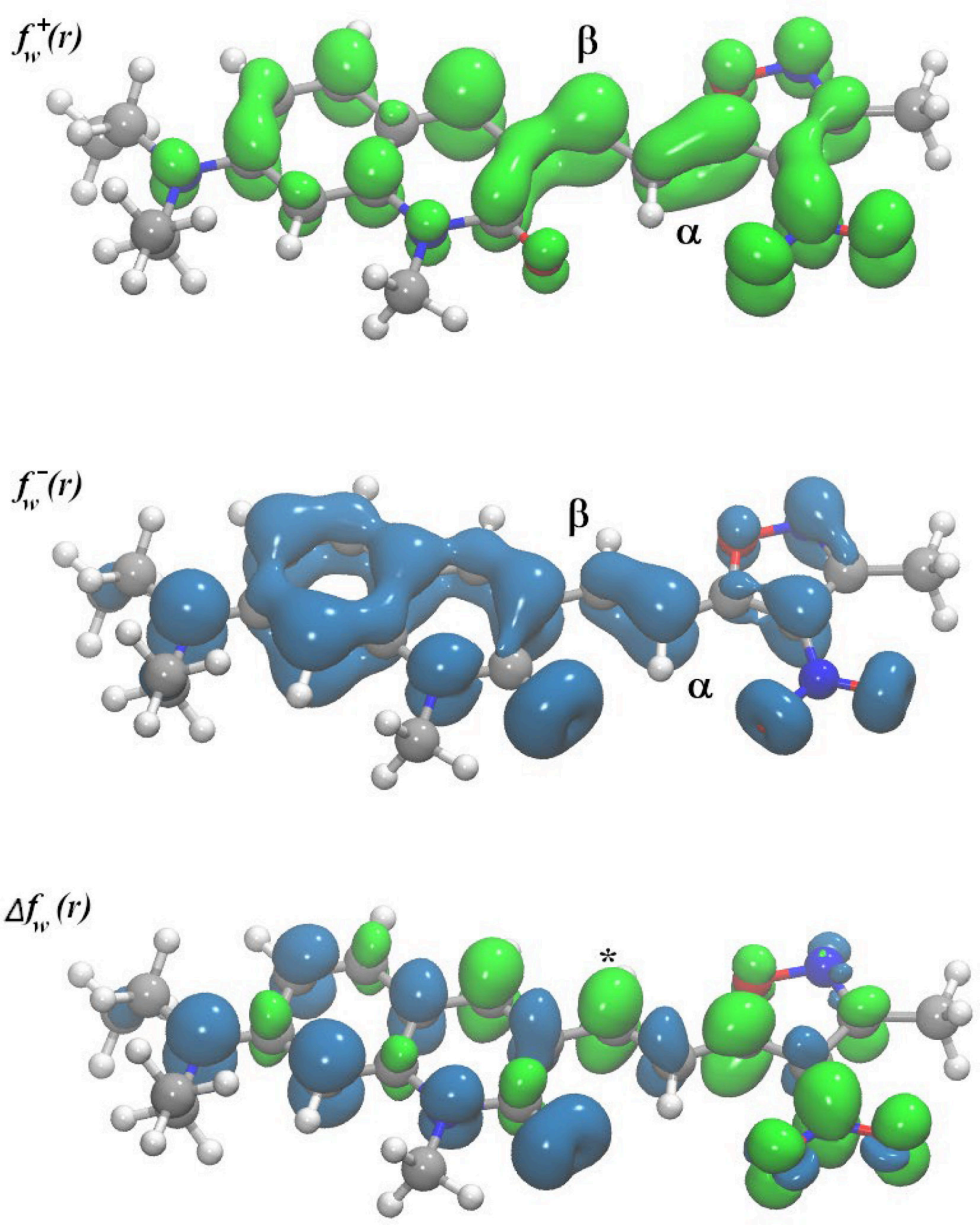
Orbital‐weighted Fukui functions, *f*_*w*_^*+*^(***r***) in green and *f*_*w*_^−^(***r***) in blue, of the two investigated **DQI** molecule obtained at the C‐PCM(1‐IodoHexaDecane)/PBE0(D3(BJ))/Def2‐TZVP level of theory. The OW Fukui dual descriptor function, Δ*f* *_w_* *(* ***r*** *)*, is also reported at the same level of theory; * highlights the electrophilic carbon atom of the *C* *_α_* = *C* *_β_* chemical group that is herein supposed to react forming the experimentally observed **DQI•**SO_3_H Michael adduct.

The reactivity proposed has been further investigated at the theoretical level by computing the molecular orbital‐averaged Fukui functions [46] which represent a rigorous generalization of frontier molecular orbital theory, indicating where a molecule is most prone to nucleophilic or electrophilic attacks (see Equations (6) and (7)). For instance, if a particular MO (e.g., HOMO) is localized on a specific atom, that atom will have a high value of $f_{w}^{-}(r)$, indicating high nucleophilicity. In this context, the best sites for nucleophilic ($f_{w}^{+}(r)$) and electrophilic ($f_{w}^{-}(r)$) attacks in the case of the **DQI** molecule in a micelle media according to the C‐PCM model [24] are shown in Figure 9. Dual descriptor reported in the lower panel, i.e., the difference ($\Delta f_{w}$), is used to directly identify nucleophilic and electrophilic centers simultaneously [46]. These C‐DFT [45] descriptors clearly highlight that *C* *_β_* features a local region with a large positive $f_{w}^{+}(r)$ or with prominent positive $\Delta f_{w}$ (green and blue isosurfaces represent positive and negative parts, respectively). Consequently, at the level of theory applied, *C* *_β_* plausibly corresponds to the site with marked electrophilic character, or equivalently, vulnerable to nucleophilic attack; in other words, the so‐called Michael acceptor modulating the reactivity observed experimentally when **DQI** interacts with HSO_3_^−^ anionic species.

**FIGURE 10:**
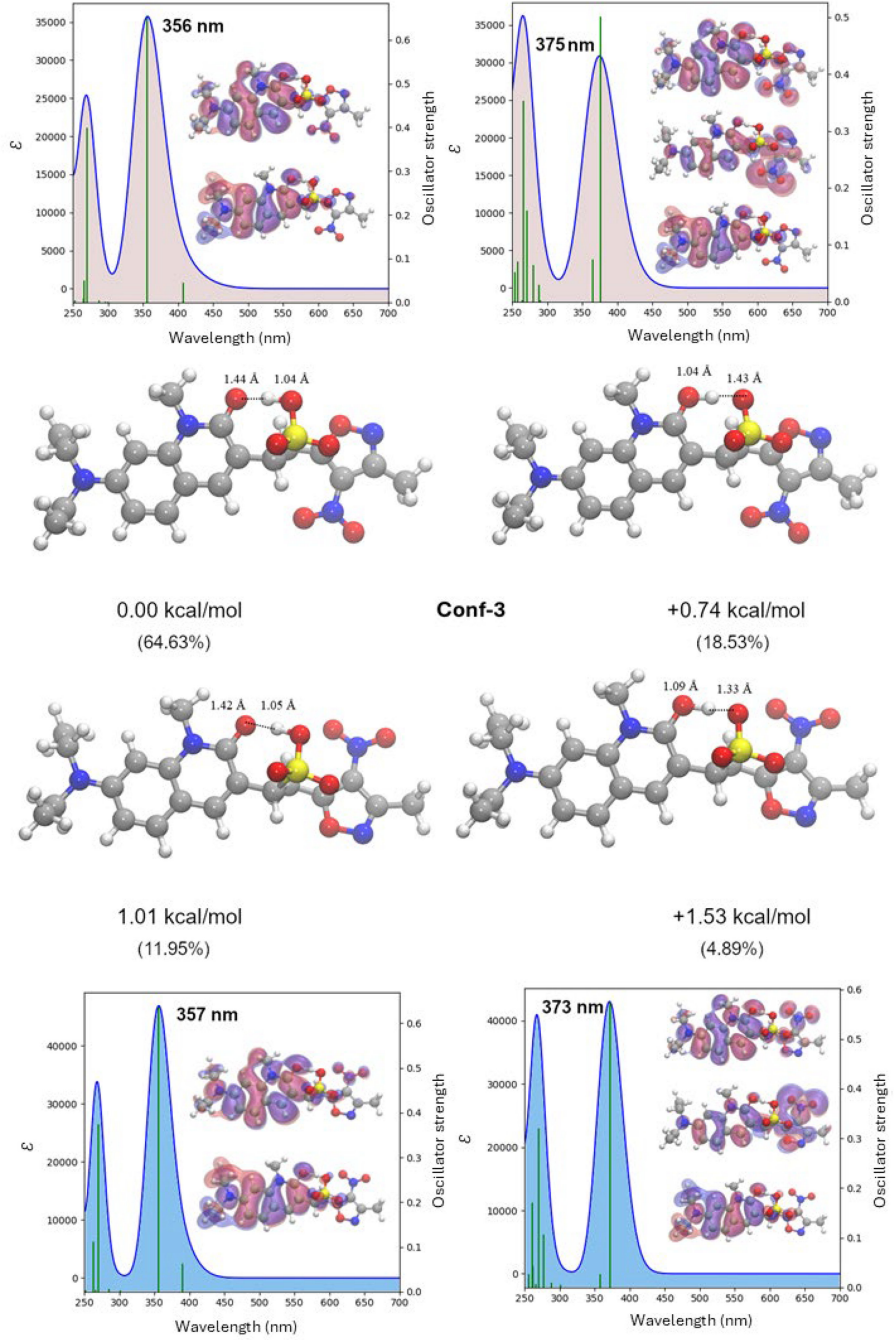
Upper panel: UV–Vis absorption spectra of different form of **DQI•**SO_3_H Michael adduct at C‐PCM/PBE0/D3(BJ)/Def2‐TZVP level. The molar absorption coefficient (*ε*) is reported in M^−1^ cm^−1^. Profiles are obtained by combining excitation energies with oscillator strengths and using Gaussian broadening functions with a FWHM of 3000 cm^−1^; oscillator strengths are reported as green histograms. An estimation of the population ratio among different species at 300 K is also reported as well as the molecular orbitals involved in the lowest energy band.

Prompted by this result, we have further extended the modeling, computing electronic excitations—at TD‐DFT level—for a subset of structures optimized at C‐PCM(1‐IodoHexaDecane)/PBE0(D3(BJ))/Def2‐TZVP level of theory. In so doing, the investigated **DQI•**SO_3_H Michael adduct is seen—taking as reference the *C* *_β_* for the thiol (nucleophile) addition—to mainly exhibit four different conformations (termed as Conf‐3 in Figure 10; Conf‐1 and Conf‐2 structures lie at higher energies, see Figure S6 in the ESI for further details) systematically showing an intramolecular H‐bond contact as shown in Figure 10. The energy difference is confined to *ca*. 1.53 kcal/mol and the most stable sampled structure—at least within the imposed approximations—shows a S─O─H─O═C intramolecular H‐bond contact featuring a distance of 1.44 Å. At + 0.74 kcal/mol, there is the associated counterpart showing a carbonyl hydrated group. Moreover, when a 180° rotation of the nitroisoxazol moiety is applied, the same species are revealed at higher energy by +1.01 and +1.53 kcal/mol. It is worth noting that these forms tend to absorb light at two distinct wavelengths; the two carbonyl hydrated forms show the lowest lying excitation band at around 374 nm while the forms with the carbonyl group are estimated to absorb photons at shorter wavelengths by *ca*. 20 nm. The band at *ca*. 374 nm arises from a combination of HOMO → LUMO and HOMO → LUMO + 1 transitions while the absorption at *ca*. 356 nm from a HOMO → LUMO + 1 excitation; these molecular orbitals are associated with transitions having a clear valence ^1^ *π* → π* character. Such a derived bimodal distribution should be plausibly useful for explaining the shape of the UV–Vis spectra following the addition of HSO_3_^−^ anion in the micellar CTABr media. As a matter of fact, as clearly shown in Figure 7a, the acquired absorption spectrum shows a maximum at around 380 nm with a shoulder at almost 360 nm. This photophysical behavior might be associated—because of these computations—to the presence of a fascinating ground‐state intramolecular proton transfer (GSIPT) process facilitated by short‐range intramolecular H‐bond contact as shown in Figure 10. In this respect, it is known in the literature that while excited‐state transfer processes are vastly more common and faster (>10^12^ s^−1^), ground‐state tautomerism can occur, especially in systems with strong internal H‐bonds [69, 70]. In this specific case, we are referring to a proton transfer from the hydroxyl oxygen (S─O─H) to the proximal carbonyl oxygen (C═O) in the observed **DQI•**SO_3_H Michael adduct through an existing intramolecular hydrogen bond. From a mechanistic point of view and taking into account the ion interchange in the Stern layer between bromide and bisulfite ions, we might initially assume that the bisulfite anion (HSO_3_^−^) attacks the *β*‐carbon of the *α*,*β*‐unsaturated Michael acceptor (the **DQI** molecular probe) located in the micellar medium, forming plausibly a carbanion intermediate. Afterward, the resulting carbanion intermediate is protonated, probably by a local water molecule, leading to the formation of the proposed final neutral Michael adduct (see Figure S7). Preliminary computations highlight that, within the simulation framework employed, upon the addition of HSO_3_^−^ the *C* *_α_* atom still maintains an sp^2^ hybridization with the negative charge being delocalized through resonance over the nitroisoxazol five‐membered ring (see Figure S8). The free energy of (HSO_3_^−^ + DQI → carbanion intermediate) reaction in 1‐IodoHexaDecane solvent as dielectric media is estimated to be slightly positive (+4.28 kcal/mol) at 298K.

**FIGURE 11:**
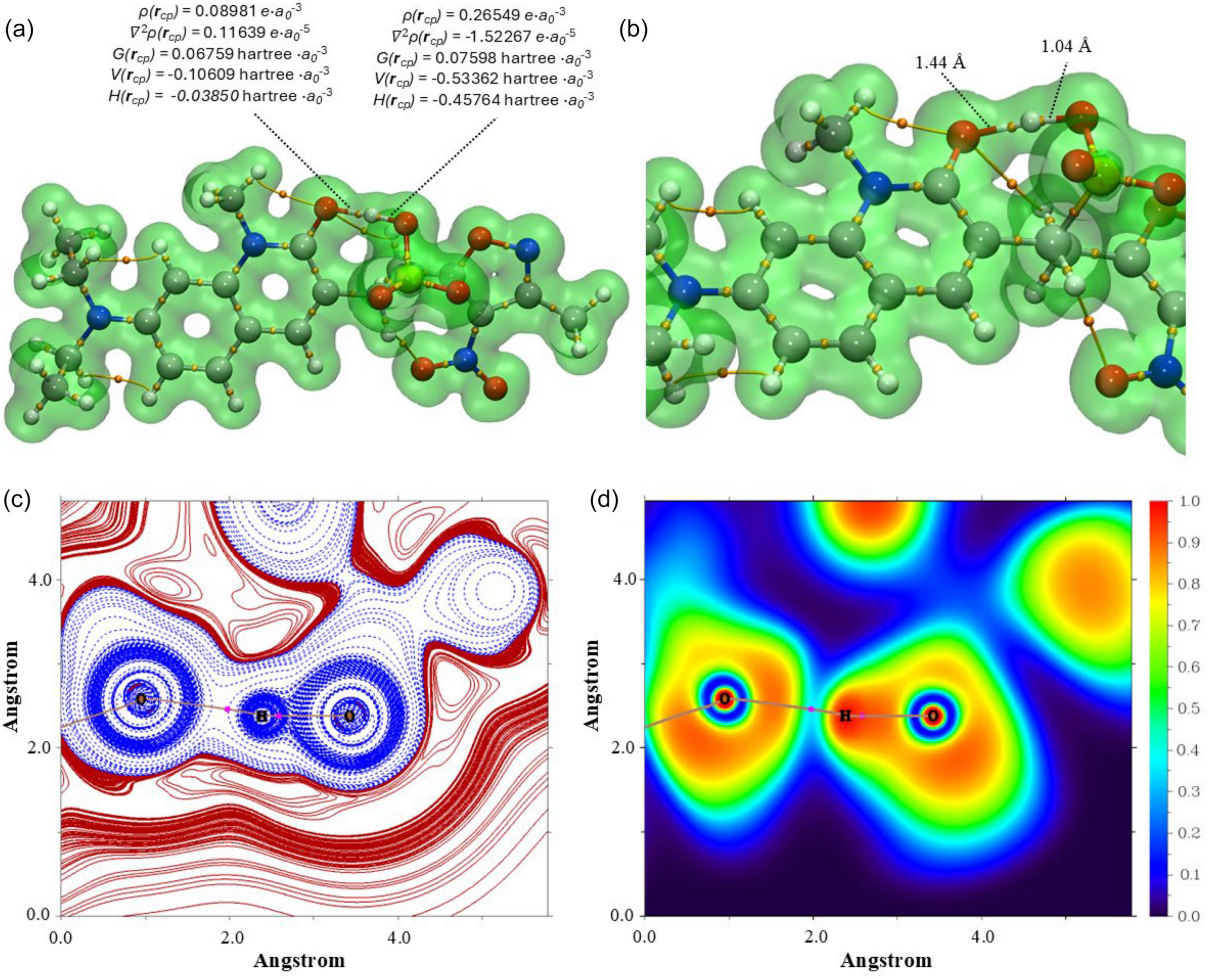
(a) and (b) 3D plot of the *H*(0, ISO) of the most stable structure sampled for the investigated **D QI•**SO_3_H Michael adduct at C‐PCM/PBE0/D3(BJ)/Def2‐TZVP level. Please note that QTAIM descriptors are also reported along the S─O─H─O═C bond pathways. (c) and (d) 2D plot of the *H*(***r***) and ELF descriptor in the plane defined by O─H─O interacting atoms. Solid/brown and dashed/blue lines correspond, respectively, to positive and negative values of the *H*(***r***); the dots in magenta indicate the CPs (+3,−1).

Such a contact is further shown in Figure 11 combining QTAIM‐based descriptors with the recently developed GLED model which allows defining the character of H‐bond contact via a graphical visualization of the *H*(0, ISO) surface extracted at QM level with explicitly correlated methods [50, 51]. The key innovation lies in the graphical visualization of *H*(*r*); the topology of the *H*(0, ISO) isosurface provides immediate insight into whether a H‐bond is: purely noncovalent, weakly covalent (wCov), strongly covalent, or partially ionic. This method is then applied to the lowest energy sampled species, at C‐PCM/PBE0/D3(BJ)/Def2‐TZVP level, and the results are shown in Figure 11a,b. The two interacting intramolecular moieties, e.g., S─O─H─O═C, overlap so to produce an outer 3D *H*(0, ISO) isosurface encompassing the nuclei of both fragments with a bonding mechanism qualitatively similar to that occurring in the formation of covalent molecules from interacting atoms. The value, at the bond critical point (BCP), of the *ρ*(*r*_cp_) = 0.08981 *e*·*a*_0_^−3^ along the S─O─H─O═C bond path (BP) is then used for estimating, via a linear regression, a dissociation energy (DE) of *ca*. 20.35 kcal/mol [51]; as a consequence, such a ground state intramolecular H‐bond contact may be characterized as a wCov interacting pattern. This trend is also confirmed by an inspection of the other derived QTAIM descriptors: When $\nabla^{2}$ *ρ*(*r*_cp_) and *H*(*r*_cp_) are both positive, the H‐bond is classified as weak and purely electrostatic; when these descriptors are both negative, the H‐bond results particularly strong with a covalent character. In contrast, a positive $\nabla^{2}$ *ρ*( *r*_cp_) coupled with a negative *H*(*r*_cp_) characterizes a moderately strong H‐bond with a partially covalent nature. The negative value of *H*(*r*_cp_) ≈ −0.038 Hartree·*a*_0_^−3^ suggests that the S─O─H─O═C interaction pattern is actually controlled by a local excess in the potential energy density, *V*(*r*_cp_), rather than by the kinetic energy density, *G*(*r*_cp_). The 2D plot of the *H*( *r*) function over the σ[O─H─O] nanoscale plane—with contour lines following the pattern ±*k* × 10^n^ (*k* = 0,1,2,4,8; *n* = −5 to 6) according to previous studies [36, 47, 71, 73]—highlights that the BCPs (shown as magenta dots) along the S─[O─H─O]═C bond paths are clearly immersed in a continuous region of negative values of *H*(*r*), see Figure 11c; moreover, this trend is further supported by the electron localization function (ELF) [74, 75] plot over the same plane reported in panel d. The ELF is a dimensionless scalar field (ranging from 0 to 1) used to describe how localized electrons are in space. A graphical representation of this function then provides a qualitative picture of the type of bonds in a molecular system and the local regions of the space where it is possible to find electron pairs. At the BCP connecting the S─[O─H─O]═C atoms, the ELF features a value at around 0.4, indicating a moderate probability of finding localized electrons. All these data support the presence of an intriguing S─O─H─O═C H‐bond network finely modulated by nanosolvation pathways and thermal fluctuations in the investigated systems. A potential energy surface scan calculation supports the derived picture shown in Figure 11. As shown in Figure S9, the hypothesized GSIPT between the two most stable conf3 species shown in Figure 10 may occur in both directions at room temperature condition (highest potential energy barrier within 0.8 kcal/mol).

## 4 Conclusion

In this study, we have developed a micelle‐assisted strategy to probe the reactivity of bisulfite anion toward a quinolinone‐based dye (**DQI)** in aqueous media. The intrinsically weak fluorescence of **DQI** was significantly enhanced in the presence of CTABr, accompanied by a marked hypsochromic shift, highlighting the crucial role of the cationic surfactant in modulating the photophysical properties of the probe. The micellar environment provided by CTABr promoted the reaction by increasing the effective solubility and local concentration of the hydrophobic dye within the Stern layer, thereby facilitating nucleophilic attack by bisulfite. Combined photophysical and NMR analyses confirmed the formation of thiol‐based Michael adducts, while DFT calculations offered molecular‐level insight into the preferred reactive site, supporting the experimental observations. Overall, this work demonstrates that CTABr micelles serve as an efficient reaction medium that not only enhances fluorescence response but also directs the formation of a **DQI•**SO_3_H Michael adduct. These findings provide a deeper understanding of micelle‐mediated reactivity and establish a foundation for the rational design of surfactant‐assisted fluorescent probes for bisulfite detection and related applications.

## Author Contributions

**Guillermo E. Quintero:** conceptualization, investigation, data curation, formal analysis, writing – original draft. **Kevin Droguett:** data curation, formal analysis, writing – original draft**.** **Constantino Zazza:** investigation, data curation, methodology, writing – original draft. **Margarita E. Aliaga:** formal analysis, resources, methodology, writing – review, and editing.

## Conflicts of Interest

The authors declare no conflicts of interest.

## Supporting information

The data that support the findings of this are available in the Supporting Information of this article: Time‐dependent absorbance profile of DQI (26 mM) monitored at 474 nm in aqueous solution (1% DMSO, pH 5.5) at 25 °C; Time‐dependent absorbance profile of DQI (26 mM) monitored at 511 nm in aqueous micellar solution of CTABr (10 mM, 1% DMSO, pH 5.5) at 25 °C; XYZ coordinates of the investigated **DQI** molecular probe and of the associated **DQI•**SO_3_H Michael adduct.

## Data Availability

The data that support the findings of this study are available in the Supporting Information of this article.
