# Supplementary material for: Thiol–Michael Addition to a Quinolin‐2‐One–Isoxazole Dye in Cationic CTABr Micellar Media: Insights From Experiments and Theory
Source: ChemistryOpen. 2026 Aug 9;15(8):e70275. doi: 10.1002/open.70275 (PMC13453944; doi:10.1002/open.70275)
Supplement: Supplementary file 1 [file OPEN-15-e70275-s001.pdf]

Electronic Supporting Information

Thiol–Michael Addition to a Quinolin-2-one–Isoxazole Dye in Cationic CTABr Micellar Media: Insights from Experiment and Theory.

Guillermo E. Quintero,[a], [b] Kevin Droguett,[a] Costantino Zazza*[c], Margarita E. Aliaga*[a]

[a] Dr. Guillermo E. Quintero, Dr. Kevin Droguett, Prof. Margarita E. Aliaga
Facultad de Química y de Farmacia, Escuela de Química, Pontificia Universidad Católica de Chile, Casilla 306, Santiago 6094411, Chile.
E-mail: mealiaga@uc.cl

[b] Dr.Guillermo E. Quintero
Department of Organic, Faculty of Chemistry and Biology, Chemistry, Universidad de Santiago de Chile, 9170022 Santiago, Chile.

[c] Dr. Costantino Zazza
Department for Innovation in Biological, Agro-food and Forest systems, Università della Tuscia (DIBAF), L.go dell'Università, s.n.c., 01100 Viterbo, Italy.
E-mail: costantino.zazza@unitus.it

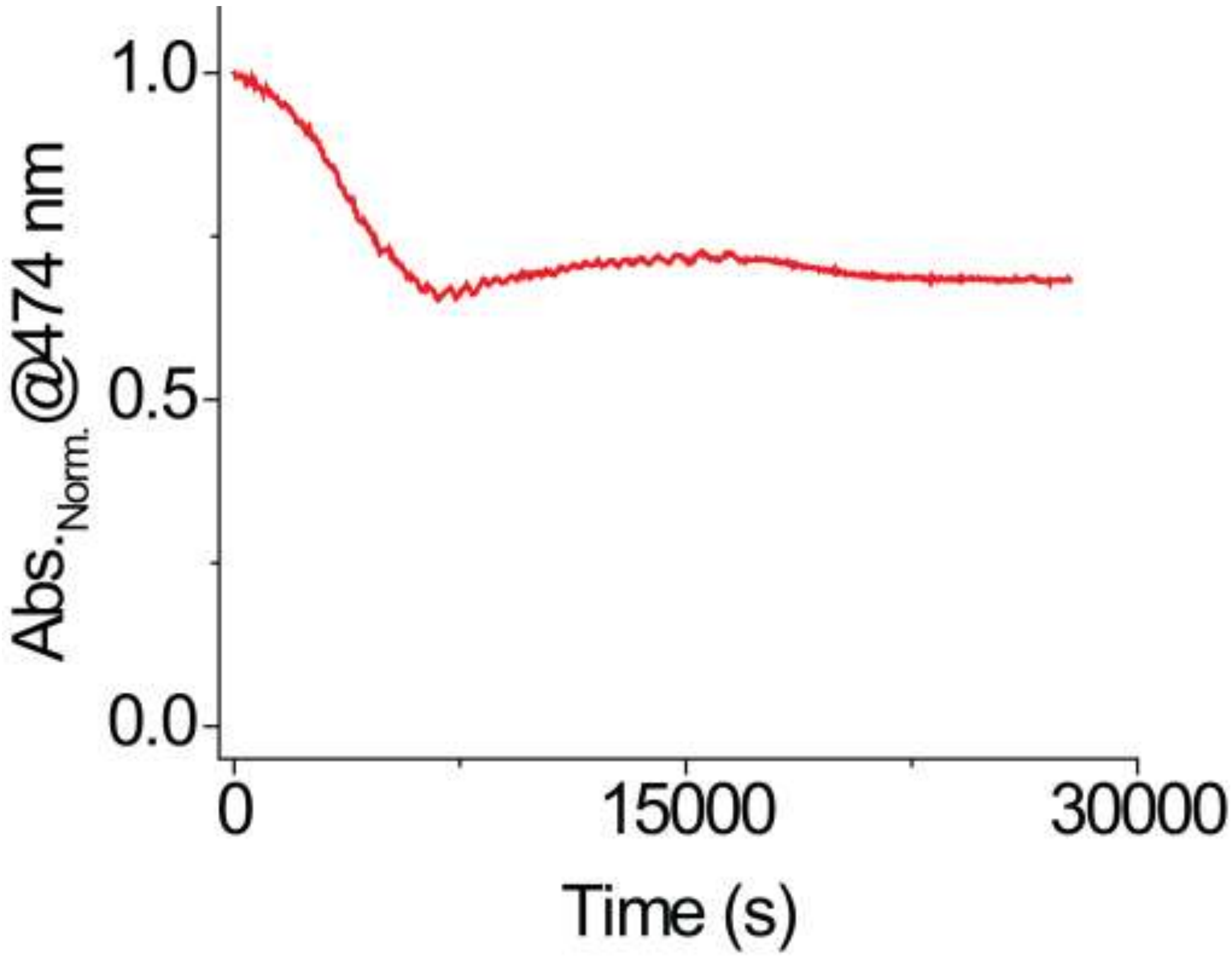

Figure S1. Time-dependent absorbance profile of DQI (26 μM) monitored at 474 nm in aqueous solution (1% DMSO, pH 5.5) at 25 °C.

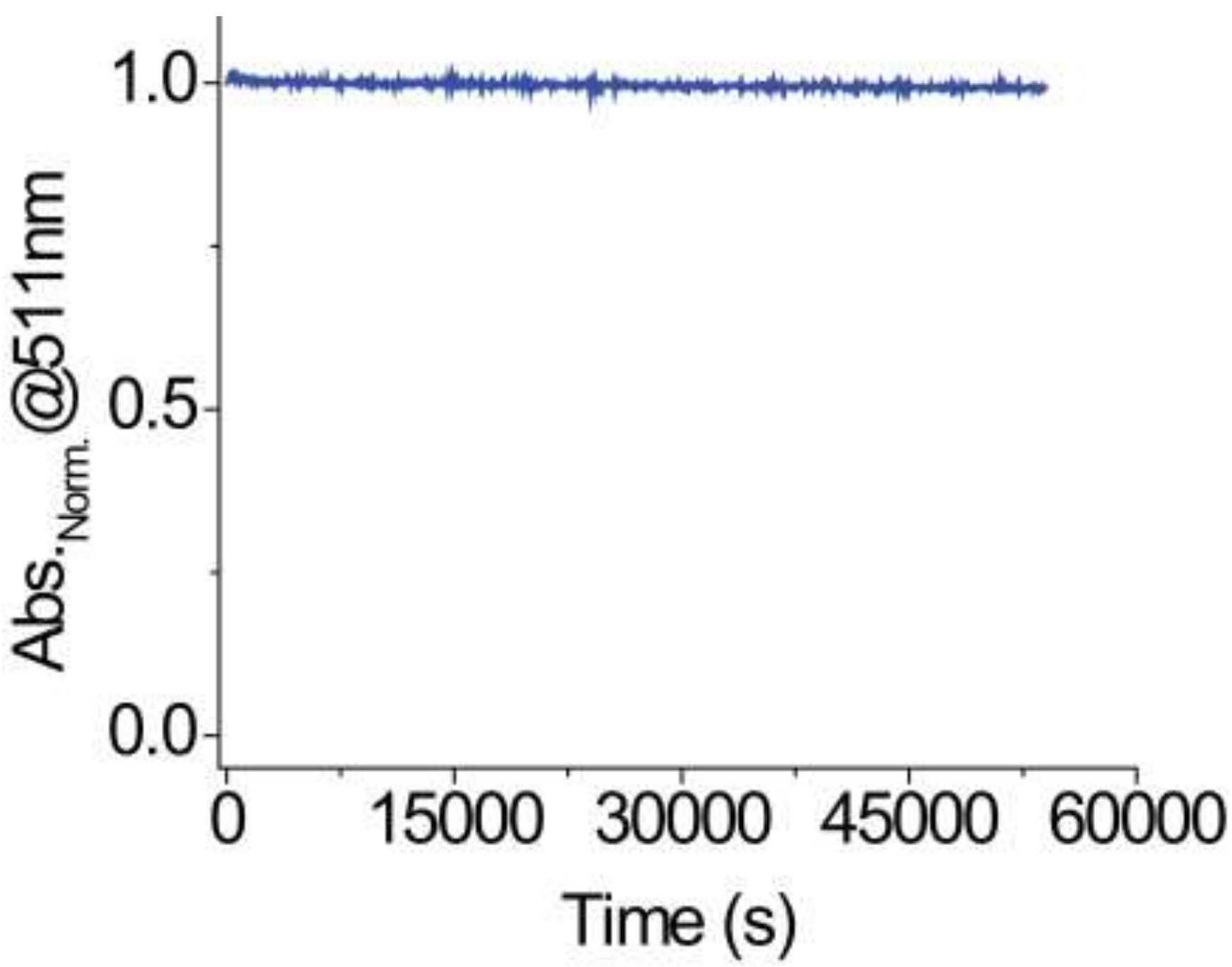

Figure S2. Time-dependent absorbance profile of DQI (26 μM) monitored at 511 nm in aqueous micellar solution of CTABr (10 mM, 1% DMSO, pH 5.5) at 25 °C.

Optimized coordinates of the **DQI** molecular probe as obtained at the C-PCM(H_2O)/PBE0(D3(BJ))/Def2-TZVP level of theory.

50

N	-1.372620	-1.326912	-0.100875
C	-1.780634	-2.715224	-0.215122
C	-0.004701	-1.062166	-0.085065
O	0.800494	-1.985401	-0.164420
C	0.389197	0.339326	0.028942
C	1.758584	0.748759	0.058150
C	2.876542	-0.017277	-0.009188
C	4.176721	0.553466	0.037198
C	5.452334	0.013400	-0.008154
N	5.798342	-1.344287	-0.121750
O	4.904312	-2.175442	-0.196456
O	6.987759	-1.630340	-0.140480
C	6.348360	1.116000	0.085391
C	7.829137	1.143434	0.088584
N	5.671272	2.221442	0.177982
O	4.322265	1.874165	0.148318
C	-0.588137	1.306204	0.114488
C	-1.948304	1.000670	0.097780
C	-2.953942	1.983747	0.190129
C	-4.278056	1.666678	0.175167
C	-4.691133	0.307170	0.063223
N	-6.002477	-0.018847	0.043336
C	-7.049870	0.967774	0.247328
C	-7.492569	1.642818	-1.039674
C	-6.452980	-1.381513	-0.179111
C	-6.535687	-2.199631	1.099026
C	-3.694633	-0.683173	-0.029370
C	-2.347281	-0.354386	-0.014932
H	-0.885733	-3.323752	-0.283203
H	-2.357855	-3.015273	0.661609
H	-2.387895	-2.859166	-1.110767
H	1.892145	1.824291	0.147933
H	2.819596	-1.088369	-0.099105
H	8.218862	0.700634	-0.828807
H	8.175385	2.172657	0.174421
H	8.217686	0.555082	0.920716
H	-0.292125	2.347983	0.200288
H	-2.650943	3.022421	0.268126
H	-5.009103	2.459821	0.229114
H	-7.894178	0.447711	0.705660
H	-6.721017	1.704887	0.981691
H	-6.666125	2.184395	-1.504338
H	-7.860892	0.905672	-1.756737
H	-8.297661	2.352178	-0.836632
H	-7.438015	-1.322239	-0.647430

H	-5.802908	-1.865106	-0.911374
H	-5.559400	-2.280215	1.581093
H	-7.227050	-1.738619	1.807964
H	-6.894393	-3.207345	0.879314
H	-3.984976	-1.717905	-0.091983

Optimized coordinates of the **DQI** molecular probe as obtained at the C-PCM(1-IodoHexaDecane)/PBE0(D3(BJ))/Def2-TZVP level of theory.

50
generated by VMD

N	1.291000	1.260000	-0.119000
C	1.716000	2.642000	-0.241000
C	-0.080000	1.013000	-0.100000
O	-0.874000	1.946000	-0.183000
C	-0.492000	-0.383000	0.022000
C	-1.866000	-0.775000	0.056000
C	-2.975000	0.005000	-0.014000
C	-4.282000	-0.549000	0.037000
C	-5.550000	0.007000	-0.010000
N	-5.879000	1.368000	-0.130000
O	-4.975000	2.188000	-0.211000
O	-7.065000	1.670000	-0.149000
C	-6.460000	-1.083000	0.091000
C	-7.941000	-1.092000	0.096000
N	-5.797000	-2.197000	0.188000
O	-4.444000	-1.867000	0.155000
C	0.473000	-1.362000	0.112000
C	1.837000	-1.074000	0.092000
C	2.830000	-2.069000	0.188000
C	4.158000	-1.769000	0.170000
C	4.589000	-0.415000	0.050000
N	5.904000	-0.106000	0.026000
C	6.939000	-1.105000	0.234000
C	7.371000	-1.792000	-1.050000
C	6.371000	1.250000	-0.204000
C	6.466000	2.073000	1.070000
C	3.605000	0.587000	-0.047000
C	2.253000	0.276000	-0.029000
H	0.829000	3.262000	-0.311000
H	2.299000	2.940000	0.633000
H	2.324000	2.774000	-1.138000
H	-2.013000	-1.848000	0.151000
H	-2.904000	1.075000	-0.110000
H	-8.327000	-0.649000	-0.823000
H	-8.301000	-2.116000	0.188000
H	-8.321000	-0.494000	0.926000
H	0.164000	-2.399000	0.204000
H	2.514000	-3.103000	0.272000
H	4.879000	-2.571000	0.227000

H	7.790000	-0.593000	0.689000
H	6.602000	-1.834000	0.973000
H	6.537000	-2.326000	-1.510000
H	7.748000	-1.064000	-1.771000
H	8.168000	-2.511000	-0.844000
H	7.355000	1.175000	-0.673000
H	5.726000	1.737000	-0.938000
H	5.492000	2.169000	1.553000
H	7.153000	1.607000	1.780000
H	6.837000	3.075000	0.844000
H	3.908000	1.618000	-0.115000

Optimized coordinates (Conf3, see Fig. 10 in the associated manuscript) of the **DQI**•SO_3H Michael adduct as obtained at the C-PCM(1-IodoHexaDecane)/PBE0(D3(BJ))/Def2-TZVP level of theory.

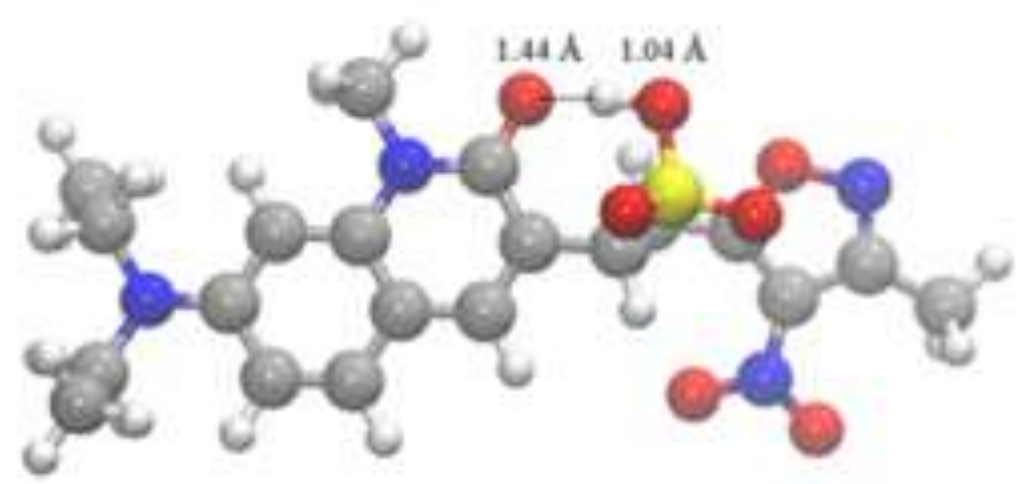

0.00 kcal/mol
(64.63%)

56

O	-4.623150	-0.985172	-1.215935
C	-3.834337	0.032581	-0.930579
C	-4.630929	1.115415	-0.676234
C	-5.970200	0.654041	-0.811949
N	-5.955678	-0.606982	-1.134881
C	-2.373788	-0.193659	-0.967894
C	-1.669330	-0.174018	0.400318
S	-2.364424	-1.383648	1.549545
O	-2.343829	-2.695162	0.723965
N	-4.192823	2.421336	-0.352885
O	-2.991513	2.612417	-0.250026
C	-7.246778	1.382369	-0.643686
C	-0.179016	-0.284939	0.271024
C	0.429844	-1.473489	-0.258546
N	1.787440	-1.474125	-0.447124
C	2.597223	-0.398061	-0.126774
C	1.997605	0.755317	0.421807
C	0.607981	0.776776	0.604683
C	2.829849	1.841272	0.745890

C	4.176974	1.801144	0.544177
C	4.791938	0.643612	-0.010987
C	3.967575	-0.445820	-0.339891
C	2.404088	-2.671627	-0.995378
O	-0.225071	-2.503531	-0.558882
N	6.129556	0.595284	-0.211657
C	6.988239	1.743666	0.017139
C	7.488551	1.841045	1.447666
O	-1.475382	-1.476123	2.674960
O	-3.740933	-1.055748	1.802581
C	6.792401	-0.611187	-0.670499
C	6.819523	-0.741947	-2.183829
O	-5.037328	3.282593	-0.199374
H	-1.897299	0.768608	0.901675
H	1.630911	-3.410412	-1.166584
H	2.898875	-2.437300	-1.938637
H	3.137234	-3.067838	-0.291792
H	-1.925920	0.612213	-1.552438
H	-2.178533	-1.135394	-1.479879
H	-7.302875	2.213926	-1.346852
H	-8.081810	0.704341	-0.811668
H	-7.312525	1.802696	0.360445
H	0.153441	1.675052	1.010983
H	2.377222	2.728757	1.173553
H	4.773108	2.655482	0.828261
H	7.833795	1.653572	-0.668496
H	6.467313	2.656452	-0.276874
H	8.151920	2.700984	1.559469
H	6.659092	1.954096	2.148374
H	8.045384	0.943282	1.724654
H	7.812360	-0.586702	-0.280776
H	6.321614	-1.483537	-0.211825
H	5.809805	-0.783832	-2.596513
H	7.334177	0.109031	-2.635048
H	7.346661	-1.652439	-2.475399
H	4.404196	-1.324776	-0.781224
H	-1.433667	-2.695611	0.202663

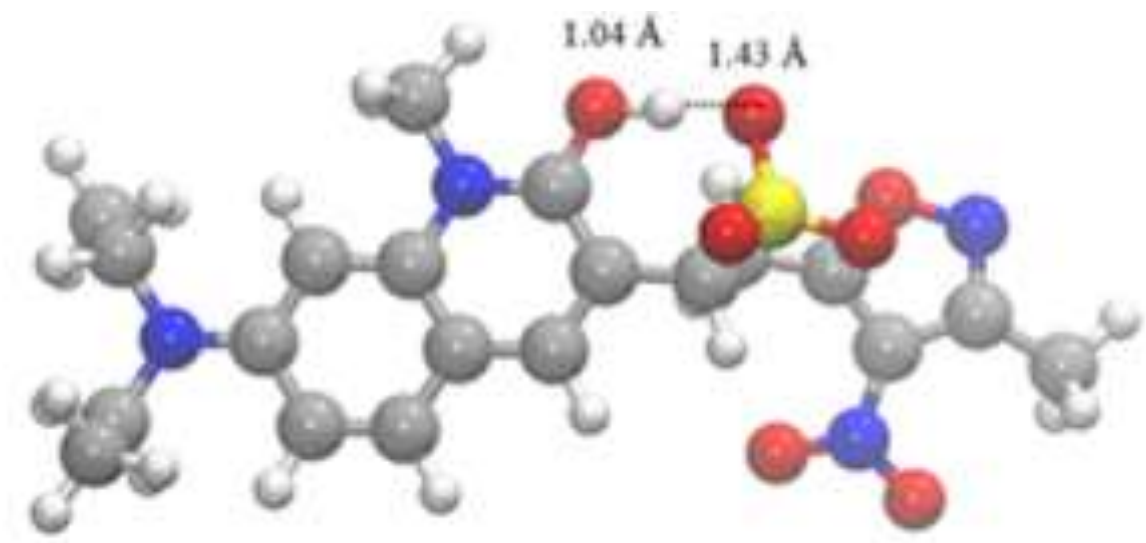

+0.74 kcal/mol
(18.53%)

56

O	-4.624737	-0.889089	-1.301792
C	-3.804597	0.079491	-0.945447
C	-4.563378	1.182681	-0.660149
C	-5.915991	0.785358	-0.856530
N	-5.945867	-0.457576	-1.241330
C	-2.353977	-0.203209	-0.961935
C	-1.692308	-0.254096	0.427371
S	-2.430592	-1.518724	1.488685
O	-2.304283	-2.766263	0.687378
N	-4.083026	2.452737	-0.267104
O	-2.875911	2.600260	-0.157381
C	-7.167362	1.557699	-0.689015
C	-0.196460	-0.363486	0.318096
C	0.447312	-1.500997	-0.224605
N	1.783882	-1.508186	-0.423403
C	2.585564	-0.424820	-0.099597
C	1.970526	0.704648	0.485848
C	0.589175	0.703244	0.676634
C	2.792022	1.799872	0.825751
C	4.132662	1.784254	0.600516
C	4.760765	0.649675	0.003670
C	3.950568	-0.446921	-0.339259
C	2.420028	-2.697098	-0.982032
O	-0.184154	-2.582832	-0.568923
N	6.091622	0.633400	-0.218942
C	6.935349	1.792599	0.023260
C	7.466592	1.856101	1.444792
O	-1.630409	-1.548171	2.693684
O	-3.814998	-1.138521	1.670895
C	6.770188	-0.548877	-0.721386
C	6.774589	-0.635467	-2.238501
O	-4.899320	3.332079	-0.063960
H	-1.913560	0.676642	0.951349
H	1.676275	-3.470294	-1.122933

H	2.874151	-2.450738	-1.942088
H	3.187687	-3.051016	-0.293992
H	-1.863570	0.597064	-1.520492
H	-2.196292	-1.139384	-1.498191
H	-7.166014	2.427613	-1.347098
H	-8.022956	0.925067	-0.920866
H	-7.250228	1.925840	0.334450
H	0.121992	1.586685	1.100144
H	2.331224	2.668715	1.281982
H	4.721002	2.640960	0.894935
H	7.765897	1.735663	-0.683935
H	6.390710	2.702855	-0.232551
H	8.116964	2.724847	1.565931
H	6.651641	1.933472	2.167419
H	8.045877	0.960898	1.681675
H	7.795491	-0.513549	-0.347046
H	6.322056	-1.441338	-0.278975
H	5.759021	-0.678591	-2.637017
H	7.272052	0.234491	-2.672802
H	7.308536	-1.530932	-2.562902
H	4.395172	-1.304854	-0.812774
H	-1.097042	-2.686763	-0.071841

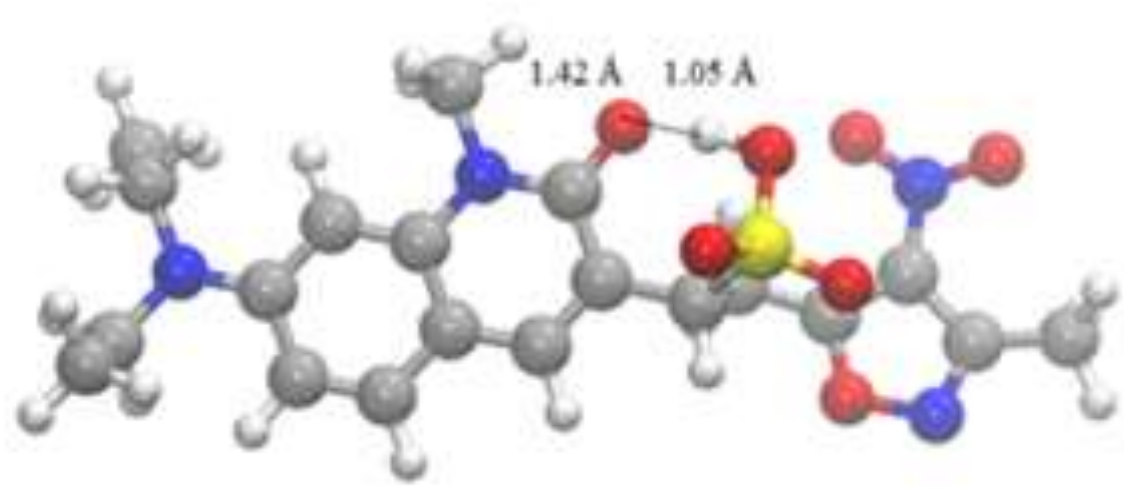

1.01 kcal/mol
(11.95%)

56

C	4.899426	0.110221	-0.985213
C	3.691605	-0.527477	-0.988969
O	3.918375	-1.822951	-1.113344
N	5.283609	-2.073523	-1.179712
C	5.878255	-0.919647	-1.096653
C	2.281650	-0.094408	-0.886163
C	1.513540	-0.738010	0.280993
S	2.285704	-0.385204	1.880189
O	3.588614	-0.983444	1.897325
C	7.355489	-0.828425	-1.131399
N	5.136127	1.507512	-0.876382
O	6.291439	1.866438	-0.748668

C	0.052284	-0.394860	0.254870
C	-0.381110	0.962698	0.445561
N	-1.718079	1.239743	0.318465
C	-2.669629	0.271380	0.048876
C	-2.244835	-1.065448	-0.098767
C	-0.875793	-1.360224	0.010045
C	-4.013573	0.601285	-0.074518
C	-4.981579	-0.381875	-0.337813
C	-4.542529	-1.727383	-0.480181
C	-3.220230	-2.041686	-0.364237
N	-6.294864	-0.061989	-0.449268
C	-6.779045	1.281602	-0.196137
C	-6.720046	2.182562	-1.419230
C	-2.151227	2.615486	0.503273
O	0.406694	1.900366	0.723456
C	-7.298542	-1.039023	-0.830249
C	-7.875313	-1.799987	0.351954
O	1.347297	-0.765324	2.896370
O	2.471073	1.145694	1.839765
O	4.183803	2.263486	-0.924404
H	-8.641006	-2.502526	0.015618
H	-8.334280	-1.113667	1.067300
H	-7.099116	-2.362143	0.875059
H	-8.094039	-0.497828	-1.348587
H	-6.880180	-1.725821	-1.568729
H	-7.811541	1.193657	0.150565
H	-6.223069	1.718420	0.637029
H	-7.323240	1.770051	-2.231195
H	-5.696057	2.290783	-1.782245
H	-7.106091	3.175665	-1.179369
H	-5.253777	-2.519387	-0.663652
H	-2.902515	-3.073267	-0.470722
H	-4.315989	1.630530	0.016217
H	-2.863404	2.673307	1.327855
H	-2.622287	2.984338	-0.409251
H	-1.282296	3.221322	0.732004
H	1.603926	1.554331	1.401556
H	-0.557086	-2.390356	-0.119087
H	1.607937	-1.825102	0.219387
H	2.248060	0.989593	-0.823270
H	1.771309	-0.392490	-1.807264
H	7.779116	-1.823262	-1.262051
H	7.678525	-0.180556	-1.947370
H	7.727149	-0.389336	-0.204783

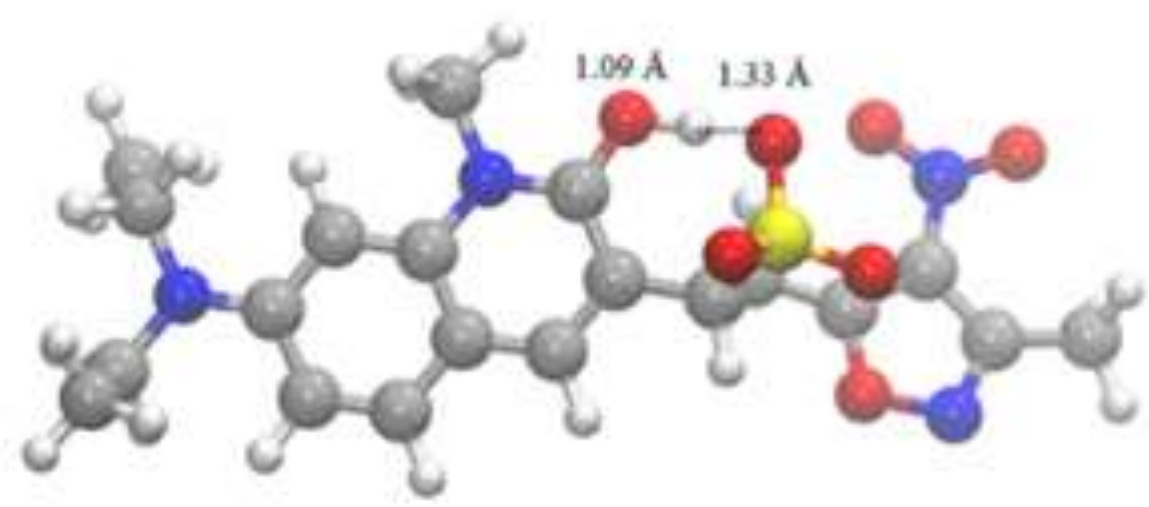

+1.53 kcal/mol
(4.89%)

56

H	-8.597444	2.571814	-0.092469
C	-7.851394	1.832206	-0.390921
H	-8.340558	1.095779	-1.032492
H	-7.084283	2.336997	-0.981557
C	-7.249966	1.164744	0.834212
H	-8.035669	0.680143	1.418855
H	-6.800368	1.903455	1.500287
N	-6.270924	0.142536	0.507018
C	-6.783793	-1.208864	0.376095
H	-7.824014	-1.129038	0.051894
H	-6.259081	-1.720130	-0.434574
C	-6.703662	-2.009082	1.665943
H	-7.277124	-1.522554	2.458102
H	-5.671755	-2.106671	2.008992
H	-7.111955	-3.010870	1.516796
C	-4.959556	0.427780	0.332431
C	-4.495884	1.775176	0.352172
H	-5.191444	2.589630	0.492151
C	-3.176617	2.060185	0.174129
H	-2.841465	3.091321	0.188564
C	-2.221428	1.047653	-0.039394
C	-2.672483	-0.288890	-0.067414
C	-4.014357	-0.589622	0.121725
H	-4.333845	-1.617435	0.125029
N	-1.739529	-1.287857	-0.292592
C	-2.203560	-2.667403	-0.374395
H	-2.957471	-2.750676	-1.157625
H	-2.634627	-2.967444	0.581499
H	-1.364950	-3.310423	-0.609513
C	-0.418979	-1.027744	-0.461696
O	0.342468	-2.039743	-0.683128
H	1.273901	-1.772267	-1.209495
C	0.060221	0.309300	-0.383675
C	-0.855956	1.307717	-0.197634
H	-0.515463	2.337332	-0.149535

C	1.527255	0.634640	-0.430866
H	1.623237	1.722110	-0.454230
S	2.316141	0.115759	-1.978656
O	1.445021	0.566092	-3.036483
O	2.343479	-1.376554	-1.855043
O	3.650609	0.661594	-1.963259
C	2.275503	0.086152	0.795424
H	2.305135	-1.000368	0.787226
H	1.724944	0.397615	1.689183
C	3.657743	0.596275	0.920818
O	3.819952	1.906810	0.927046
N	5.167129	2.230433	1.042942
C	5.814938	1.104607	1.110300
C	7.290190	1.090489	1.234452
H	7.661282	2.113087	1.288230
H	7.592810	0.541284	2.127012
H	7.734803	0.582898	0.377563
C	4.890581	0.021836	1.051055
N	5.193897	-1.364678	1.094423
O	4.274120	-2.159325	1.162592
O	6.369395	-1.678085	1.069463

Conf-1 XYZ coordinates

56

N	2.014484	1.815241	-0.067722
C	2.770986	3.048307	-0.180597
C	0.631471	1.906096	-0.121683
O	0.074029	2.996321	-0.254908
C	-0.105700	0.661123	-0.007462
C	-1.593801	0.774095	-0.082297
S	-2.177253	0.360613	-1.734070
O	-3.581956	0.651592	-1.799072
O	-1.296407	0.921140	-2.707909
O	-1.975228	-1.196569	-1.814274
H	-2.695292	-1.650166	-1.314181
C	-2.318312	-0.041553	0.999512
C	-3.783390	0.155184	1.053013
C	-4.877514	-0.618061	0.772733
N	-4.890654	-1.922314	0.233757
O	-3.847749	-2.381811	-0.234101
O	-5.929766	-2.539549	0.258649
C	-6.012849	0.176440	1.108632
C	-7.454731	-0.139011	1.006091
N	-5.610561	1.333932	1.542660
O	-4.218460	1.308775	1.518062
C	0.550204	-0.517163	0.143597

C	1.956549	-0.575288	0.195700
C	2.664032	-1.777329	0.353764
C	4.028876	-1.815675	0.397926
C	4.785790	-0.617856	0.285083
N	6.141797	-0.642666	0.323065
C	6.884306	-1.859378	0.597021
C	7.188551	-2.671116	-0.651154
C	6.937066	0.548545	0.093675
C	7.181151	1.360732	1.355363
C	4.085621	0.591370	0.129847
C	2.696737	0.621812	0.081968
H	2.066111	3.864155	-0.298559
H	3.368999	3.211561	0.718042
H	3.432391	3.009739	-1.048303
H	-1.858545	1.832016	0.001776
H	-2.106736	-1.104672	0.903644
H	-1.909370	0.287383	1.958081
H	-7.704381	-0.977949	1.656795
H	-8.041348	0.732791	1.291462
H	-7.707396	-0.431416	-0.014285
H	0.000539	-1.450301	0.218642
H	2.099790	-2.700551	0.434306
H	4.522862	-2.770894	0.500233
H	7.816628	-1.564928	1.085129
H	6.344285	-2.461739	1.330085
H	7.768647	-3.560230	-0.394274
H	6.269292	-2.991663	-1.145690
H	7.768526	-2.080451	-1.364086
H	7.891078	0.224278	-0.328883
H	6.463500	1.160810	-0.677386
H	6.241389	1.704107	1.792551
H	7.703679	0.761004	2.104182
H	7.795017	2.235723	1.130802
H	4.634976	1.515401	0.066761

Conf-2 XYZ coordinates

56

C	-2.616290	-0.373764	0.221536
N	-1.798606	-1.393912	0.669846
C	-0.417558	-1.395391	0.520219
C	0.165629	-0.242777	-0.134148
C	-0.627386	0.765043	-0.582126
C	-2.025862	0.736603	-0.420179
C	-3.993842	-0.426384	0.400804
C	-4.829816	0.609409	-0.051442

C	-4.223086	1.720552	-0.698145
C	-2.868249	1.766965	-0.866825
C	-2.399368	-2.539017	1.327607
O	0.258990	-2.340429	0.927409
C	1.646528	-0.161363	-0.310396
S	2.286632	-1.266750	-1.602225
O	1.237188	-1.602514	-2.509224
N	-6.174374	0.550379	0.120513
C	-6.825608	-0.621837	0.673634
C	-6.893931	-0.611229	2.192314
H	1.877671	0.809822	-0.755967
C	2.446138	-0.295900	0.996345
C	3.895123	-0.058175	0.836706
C	4.681371	1.048820	0.698226
C	6.016940	0.569912	0.567528
N	6.015505	-0.730040	0.620506
O	4.688680	-1.118274	0.797857
N	4.232206	2.393114	0.676210
O	5.072367	3.270550	0.636449
C	7.279596	1.320133	0.388410
O	3.028903	2.590555	0.695531
C	-7.047467	1.654536	-0.233372
C	-7.520553	1.606452	-1.676719
O	3.496774	-0.667896	-2.091966
O	2.674216	-2.618366	-0.883163
H	-1.604986	-3.229532	1.589500
H	-2.922887	-2.226464	2.233251
H	-3.106079	-3.033888	0.658633
H	2.048788	0.454551	1.680836
H	2.281006	-1.282922	1.425776
H	7.446362	1.984725	1.237017
H	8.111576	0.623310	0.299322
H	7.224839	1.942341	-0.505929
H	-0.182836	1.623647	-1.077706
H	-2.420439	2.618985	-1.367479
H	-4.826241	2.531267	-1.080161
H	-7.906564	1.615148	0.440859
H	-6.547225	2.601059	-0.019837
H	-8.192715	2.441910	-1.884648
H	-6.677798	1.662932	-2.368790
H	-8.060354	0.677631	-1.875337
H	-7.835201	-0.657737	0.257338
H	-6.323415	-1.522794	0.313346
H	-5.895219	-0.583216	2.632735
H	-7.444883	0.262951	2.546829
H	-7.404220	-1.505899	2.555685
H	-4.426306	-1.268499	0.913990
H	3.567080	-2.552642	-0.506191

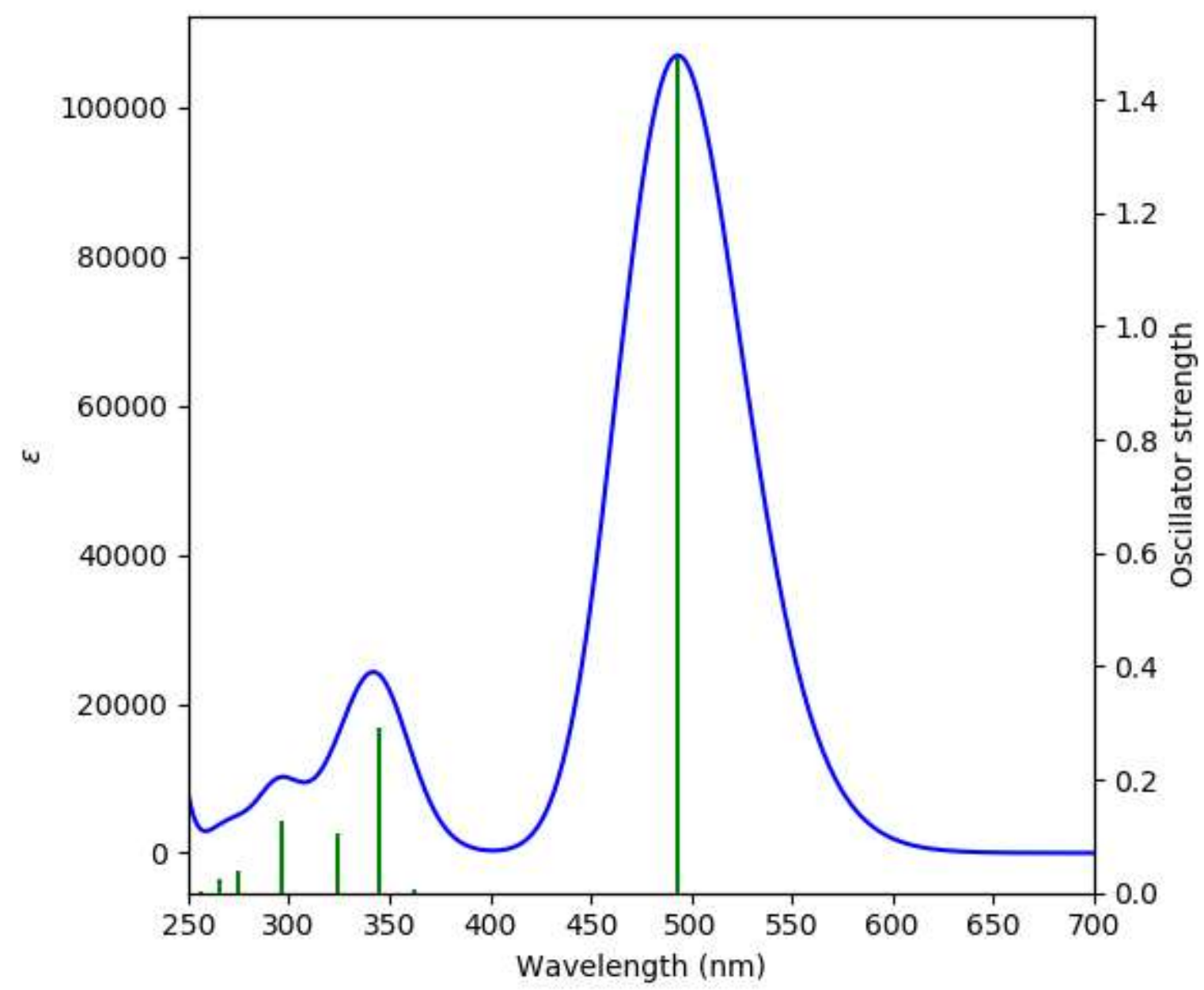

Figure S3. UV-Vis absorption spectra of **DQI** at C-PCM(1-IodoHexaDecane)/PBE0[D3(BJ)]/Def2-TZVP level. The molar absorption coefficient (Ɛ) is reported in $M^{-1}cm^{-1}$. Profiles are obtained by combining excitation energies with oscillator strengths and using Gaussian broadening functions with a full width at half maximum (FWHM) of 3000 cm^{-1}; oscillator strengths are reported as green histograms

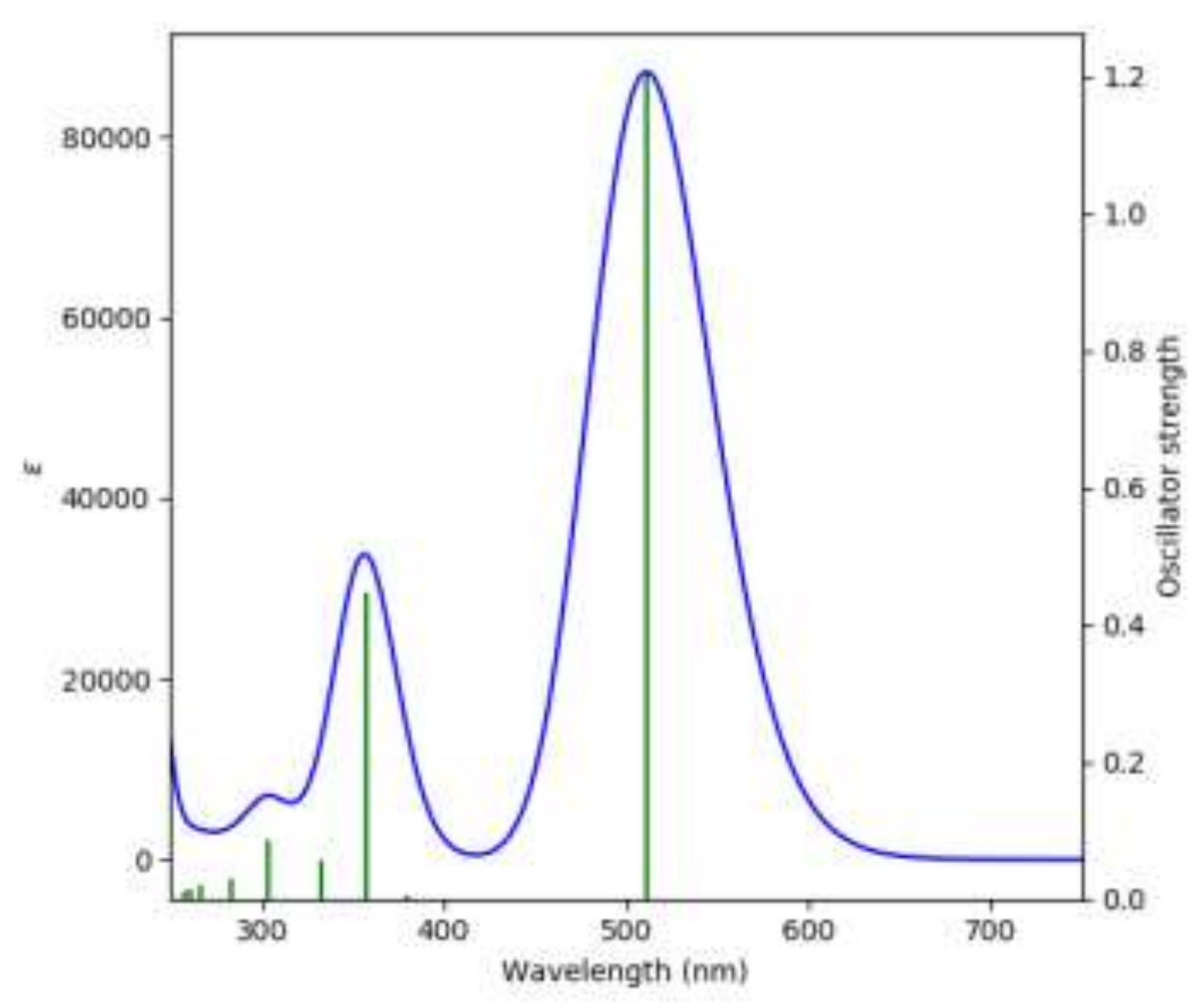

Figure S4. UV-Vis absorption spectra of a model **DQI**/CTA^+ supramolecular assembly at C-PCM(H_2O)/PBE0[D3(BJ)]/Def2-TZVP level. The molar absorption coefficient (Ɛ) is reported in $M^{-1}cm^{-1}$. Profiles are obtained by combining excitation energies with oscillator strengths and using

Gaussian broadening functions with a full width at half maximum (FWHM) of 3000 cm^{-1}; oscillator strengths are reported as green histograms

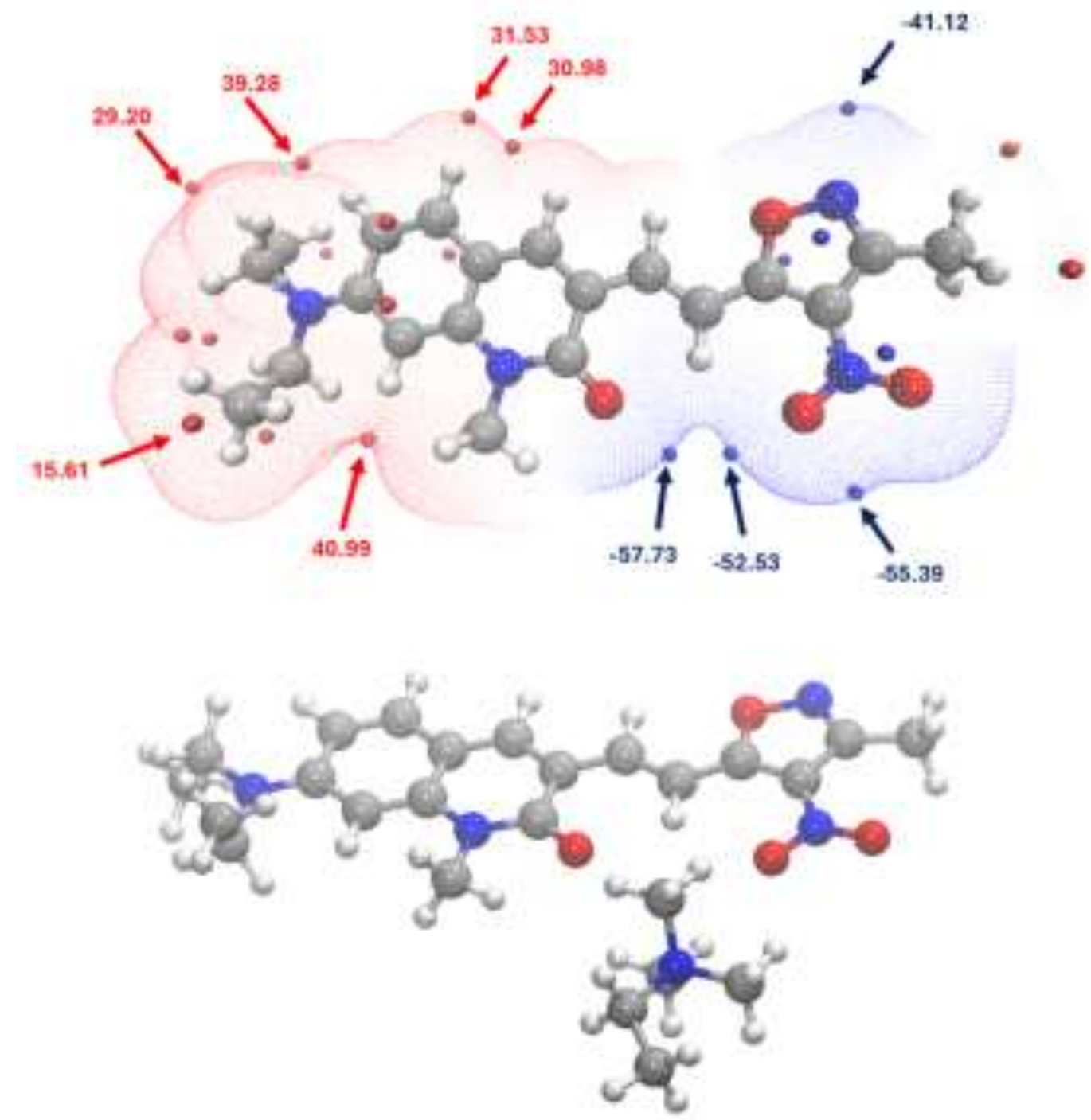

Figure S5. Upper panel: ESP (in kcal/mol) colored electron density isosurface on the vdW surface ($\rho = 0.001\ a_0 \cdot e^{-3}$) of the **DQI** molecular probe at C-PCM(H_2O)/PBE0[D3(BJ)]/Def2-TZVP level of computation. Lower panel: optimized geometry of a model **DQI**/CTA^+ supramolecular assembly in aqueous environment at C-PCM level.

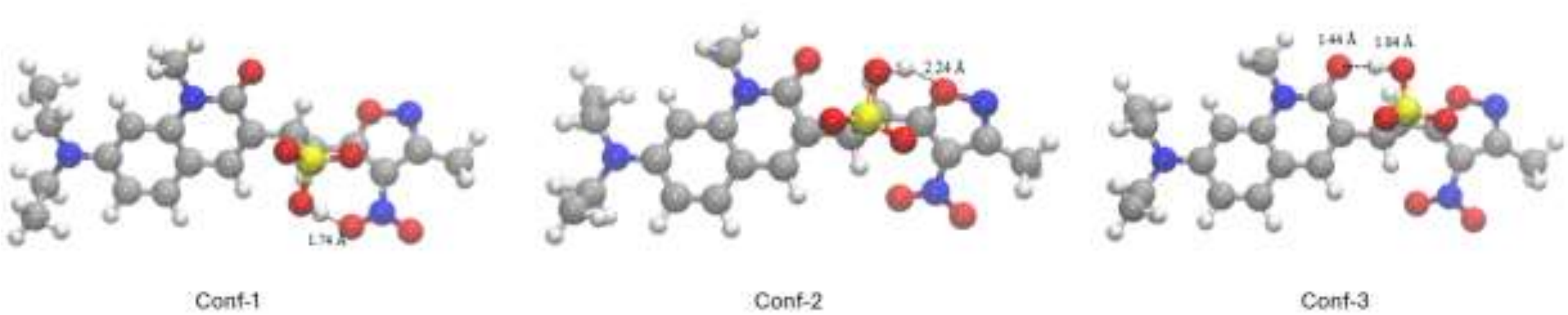

Figure S6. Optimized geometry of different conformations of the titled **DQI•**SO_3H Michael adduct as obtained at the C-PCM(1-IodoHexaDecane)/PBE0(D3(BJ))/Def2-TZVP level of theory. Relative stabilities with respect to the Conf-3 geometry (in kcal/mol): Conf1 + 6.83; Conf-2: + 10.99.

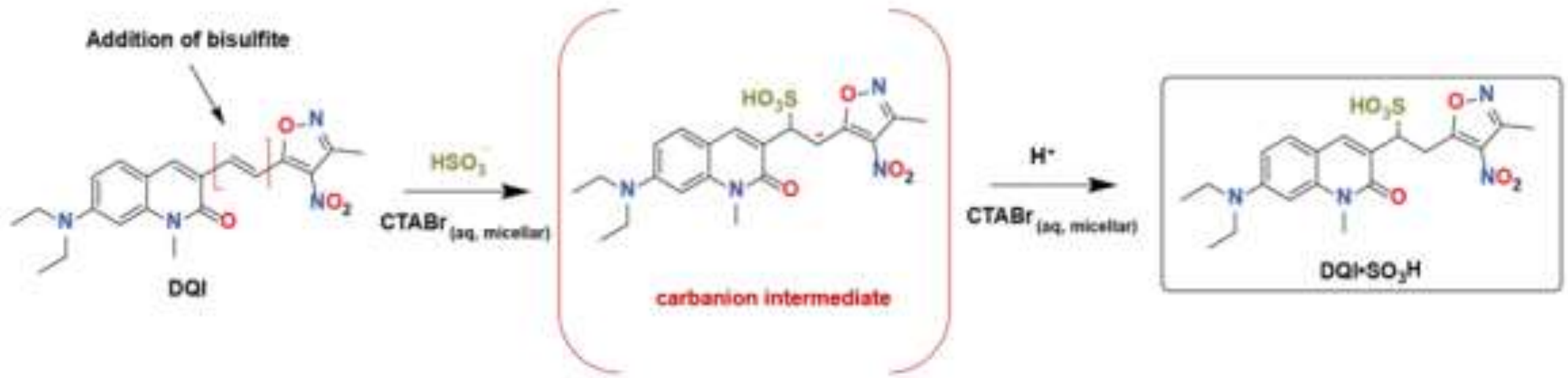

Figure S7. A plausible carbanion intermediate connecting, via Thiol-Michael addition, the **DQI** with the simulated **DQI**·HSO_3 adduct.

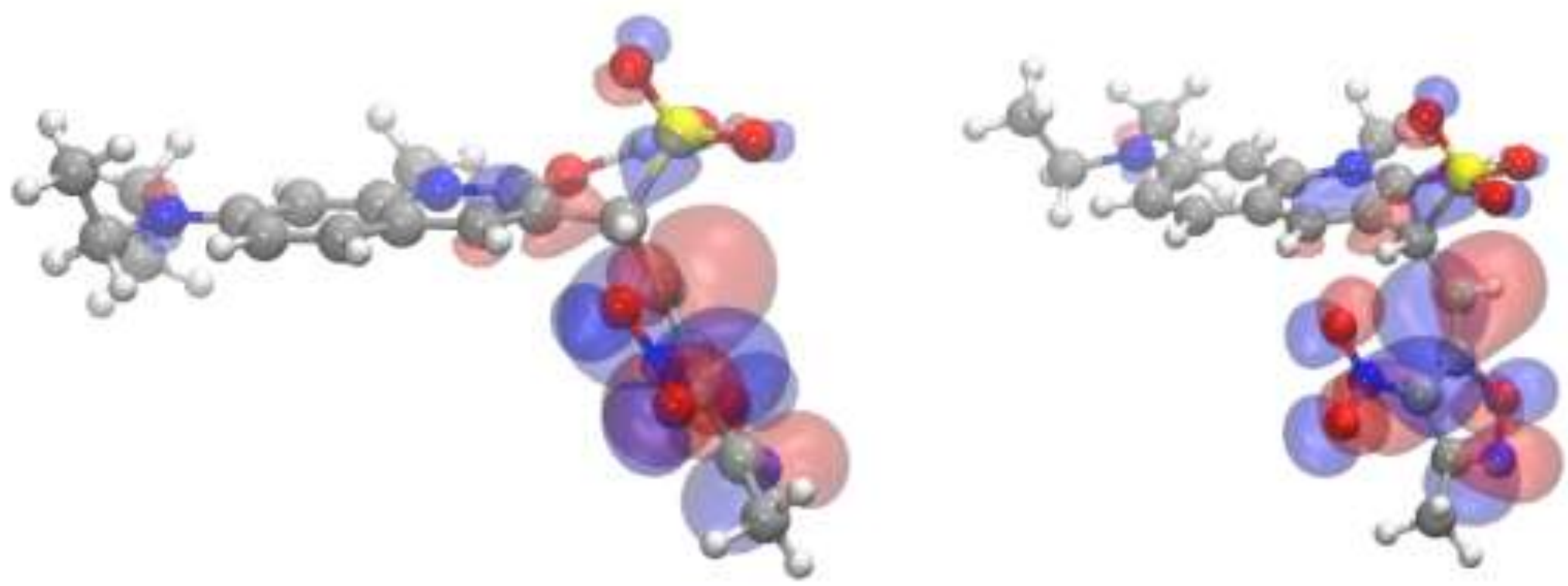

Figure S8. Two different orientations showing the HOMO molecular orbital estimated – at C-PCM(1-iodohexadecane)/PBE0[D3(BJ)]/Def2-TZVP level - for the carbanion intermediate species reported in Figure S7; the blue color reflects the positive part of the corresponding eigenvector; an isodensity value of 0.015 $e{\cdot}a_0^{-3}$ is used.

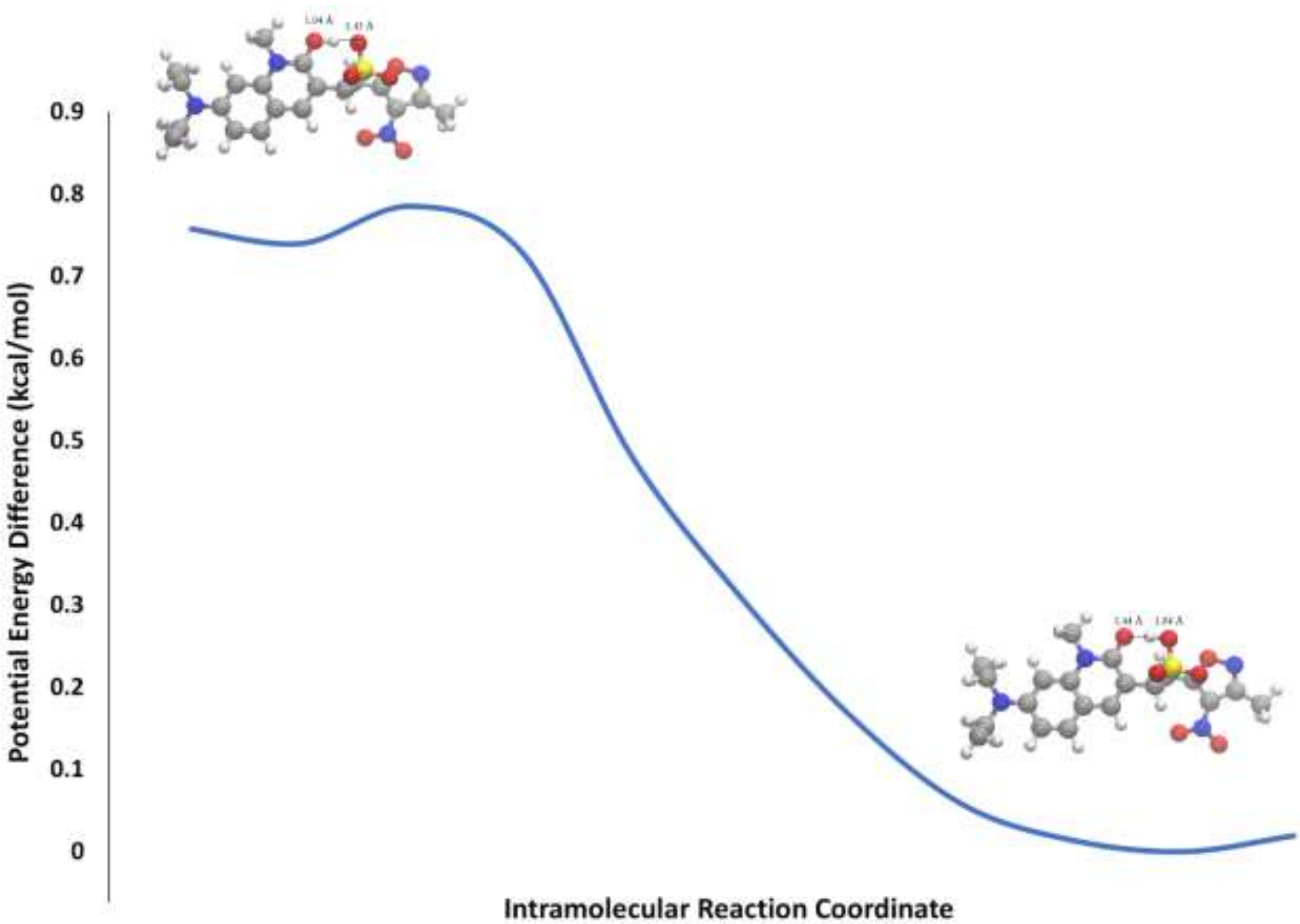

Figure S9. Relaxed Potential Energy Surface (PES) scan modulating a ground state intramolecular proton transfer (GSIPT) within the titled **DQI•**SO_3H Michael adduct as obtained at the C-PCM(1-IodoHexaDecane)/PBE0(D3(BJ))/Def2-TZVP level of theory. Relative stabilities with respect to the lowest Conf-3 tautomeric forms are those reported in Figure 10.
